# Assessing Performance of Spore Samplers in Monitoring Aeromycobiota and Fungal Plant Pathogen Diversity in Canada

**DOI:** 10.1128/AEM.02601-17

**Published:** 2018-04-16

**Authors:** Wen Chen, Sarah Hambleton, Keith A. Seifert, Odile Carisse, Moussa S. Diarra, Rick D. Peters, Christine Lowe, Julie T. Chapados, C. André Lévesque

**Affiliations:** a Ottawa Research and Development Centre, Science and Technology Branch, Agriculture and Agri-Food Canada, Ottawa, Canada; b Saint-Jean-sur-Richelieu Research Development Centre, Science and Technology Branch, Agriculture and Agri-Food Canada, Saint-Jean-sur-Richelieu, Québec, Canada; c Guelph Research and Development Centre, Science and Technology Branch, Agriculture and Agri-Food Canada, Guelph, Ontario, Canada; d Charlottetown Research and Development Centre, Science and Technology Branch, Agriculture and Agri-Food Canada, Charlottetown, Prince Edward Island, Canada; University of Tennessee and Oak Ridge National Laboratory

**Keywords:** air- and rain-borne fungi, high-throughput sequencing, HTS, metabarcodes, plant fungal pathogens, spore sampler

## Abstract

Spore samplers are widely used in pathogen surveillance but not so much for monitoring the composition of aeromycobiota. In Canada, a nationwide spore-sampling network (AeroNet) was established as a pilot project to assess fungal community composition in air and rain samples collected using three different spore samplers in the summers of 2010 and 2011. Metabarcodes of the internal transcribed spacer (ITS) were exhaustively characterized for three of the network sites, in British Columbia (BC), Québec (QC), and Prince Edward Island (PEI), to compare performance of the samplers. Sampler type accounted for ca. 20% of the total explainable variance in aeromycobiota compositional heterogeneity, with air samplers recovering more Ascomycota and rain samplers recovering more Basidiomycota. Spore samplers showed different abilities to collect 27 fungal genera that are plant pathogens. For instance, Cladosporium spp., Drechslera spp., and Entyloma spp. were collected mainly by air samplers, while Fusarium spp., Microdochium spp., and Ustilago spp. were recovered more frequently with rain samplers. The diversity and abundance of some fungi were significantly affected by sampling location and time (e.g., Alternaria and Bipolaris) and weather conditions (e.g., Mycocentrospora and Leptosphaeria), and depended on using ITS1 or ITS2 as the barcoding region (e.g., Epicoccum and Botrytis). The observation that Canada's aeromycobiota diversity correlates with cooler, wetter conditions and northward wind requires support from more long-term data sets. Our vision of the AeroNet network, combined with high-throughput sequencing (HTS) and well-designed sampling strategies, may contribute significantly to a national biovigilance network for protecting plants of agricultural and economic importance in Canada.

**IMPORTANCE** The current study compared the performance of spore samplers for collecting broad-spectrum air- and rain-borne fungal pathogens using a metabarcoding approach. The results provided a thorough characterization of the aeromycobiota in the coastal regions of Canada in relation to the influence of climatic factors. This study lays the methodological basis to eventually develop knowledge-based guidance on pest surveillance by assisting in the selection of appropriate spore samplers.

## INTRODUCTION

In agricultural systems, many crop diseases are initiated by spores of fungal pathogens (1), with air dispersal as one of the major mechanisms for spreading disease to surrounding plants (2) or over long distances, with spores remaining viable and causing outbreaks far from the source location. The use of spore samplers, especially a network of samplers at the regional scale, to directly measure air- and rain-borne inocula of fungal pathogens of crops from large, distant sources, can serve as a disease-forecasting system and is an effective way to support early warning of disease risk and to control epidemics (1, 3–7). One example of long-distance dispersal was the introduction of Asian soybean rust (Phakopsora pachyrhizi) to the United States (7), most likely by hurricane Ivan in 2004, and it was later carried to southeast Canada by prevailing winds in subsequent growing seasons as the disease became established in southern states (6). Isard et al. (7) utilized a network of rainfall samplers and developed an aerobiological model to forecast the timing of the northward spread of P. pachyrhizi, which was validated by monitoring incursions of the disease in North America through sentinel plot observations and species-specific molecular diagnosis of DNA extracts from the spore samplers. Rainfall collectors also may be useful for early warning of other fungal pathogens whose life cycle, viability, and dispersal are strongly associated with rainfall (8–11). For example, the slimy, asexual conidia of Fusarium spp. (12), Phoma exigua, and Pyrenopeziza brassicae (13, 14), as well as the zoospores of pathogens in the kingdom Stramenopiles, such as Phytophthora, Peronospora, and Pythium spp. (14, 15) (16), are often splash dispersed although these fungi may be aerially dispersed as sexual spores (ascospores or oospores) in other morphs of the same life cycles. Under extreme climatic conditions, e.g., strong wind and rain, some splash-dispersed pathogens can be disseminated over long distances (17).

Pathogens like Puccinia spp., Uromyces phaseoli, Ustilago spp., Erysiphe spp., and Drechslera spp. (16, 18) are produced in dry chains, with spores projecting above the host surface, and thus are dispersed by air currents (13). Such spores can be captured effectively by active volumetric samplers, which usually can quantify the amount of spores per volume of air but need to operate for an extended time for the detection of relatively rare spores, such as those of crop pathogens (1, 19). Installing samplers just above the crop canopy (1) and increasing the number of samplers for a larger spatial scale of monitoring may reliably monitor airborne inocula (3, 20). In one study, Carisse et al. (3) used rotating-arm air samplers to monitor the aerial conidium concentration of Botrytis leaf blight (Botrytis squamosa) and the relationship with lesion density on onion. Passive spore samplers, a third category, collect gravitational deposition from the atmosphere from both air and rain. These types of samplers are easy to operate, economical, and energy efficient (1) but do not allow calculation of spore concentrations in air as active air samplers do. All kinds of samplers may also capture fungal spores disseminated by invertebrates (19).

The choice and optimal sampling conditions (location and height) of ground-based samplers should consider the aerodynamics of the targeted fungal spores, the surrounding vegetation, meteorological conditions of sampling sites, and the desired duration of sampling (21). In particular, both rain and wind can significantly affect the dispersal and viability of all fungal spores, irrespective of which dispersal mechanism they exploit. Wind aids aerosol dispersal of spores that can be carried inside or on the surfaces of rain droplets, while rain facilitates the deposition of dry particles and provides the free water required for germination and infection. Rainfall events also provoke the detachment of spores from the host and facilitate the deposition of spores that pass through the upper atmosphere. Therefore, total precipitation during the collecting cycle can significantly affect deposition. Some climatic conditions, e.g., humidity and air temperature, affect not only the production and release of pathogen inoculum but also the susceptibility of the host (22). Other environmental conditions, such as solar and UV radiation, turbulent diffusion, and wind shear during transport, also contribute to the dispersal of fungal spores (23).

From 2007 to 2013, the Agriculture and Agri-Food Canada (AAFC) network of air and rain samplers in five provinces monitored the south-to-north pattern of Asian soybean rust migration toward Canada using molecular probes (6). Using this approach, it was possible to detect spores of P. pachyrhizi in Canadian air well before the first and only confirmation of an infected soybean plant in Ontario (6). In recent years, high-throughput sequencing (HTS)-based approaches, including metabarcoding, shotgun metagenomics, and metatranscriptomics, demonstrated sensitive and broad taxonomic and functional coverage, which allows researchers to characterize total microbial diversity and to monitor the distribution and fluctuation of plant pathogens even at low concentrations. The metabarcoding approach utilizes DNA markers to generate a data matrix that is less complex than that using shotgun metagenomics and is popular for microbial community profiling in biodiversity studies. The objectives of this study were to generate broad-spectrum temporal and spatial baseline air- and rain-borne fungal profiles at agricultural sites in three Canadian locations (Agassiz, British Columbia [BC]; Saint-Clotilde, Quebec [QC]; and Harrington, Prince Edward Island [PEI]) using a metabarcoding approach and to evaluate the performance of the three types of spore samplers in detecting specific taxonomic groups, e.g., genera containing plant pathogens, under different meteorological conditions. Our goal was to demonstrate that spore sampling combined with HTS can be an effective approach for agricultural pest surveillance and that pathogen dispersal pathways can be monitored and inoculum potential can be determined before infected plants are found.

## RESULTS

### Summary of the metabarcodes.

We obtained more than 6.7 million raw 454 pyro-tagged internal transcribed spacer (ITS) sequences with sequence lengths ranging from 325 to 725 bp. A total of 4.7 million sequences passed the quality-trimming process, with an approximately equal number of amplicons being sequenced from the ITS1 region using the ITS5 5′ primer and the ITS2 region using the ITS4 3′ primer. After chimeras and singleton operational taxonomic units (OTUs) were removed, 8,610 OTUs (referred to as ITS1_OTU) containing 1.57 million forward sequences (mainly ITS1 and 5.8S) and 8,013 OTUs (referred to as ITS2_OTU) containing 1.7 million reverse sequences (mainly 5.8S and ITS2) remained. One sample (identification [ID] code YE151W13SC_P) was removed from the downstream analyses because of low sequence count (<1,000 reads).

### Putative species diversity based on different ITS regions.

**FIG 1:**
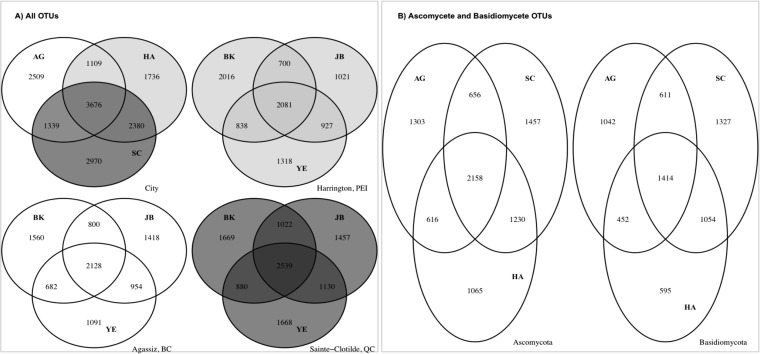
(A) OTUs detected at different sampling sites and by different spore samplers. Less than one-fifth of the total OTUs can be found at both the eastern (Harrington and Sainte-Clotilde) and western (Agassiz) coasts of Canada. The two eastern locations shared two times more OTUs than either eastern location shared with the western location. At each of the sampling locations, more OTUs were found by both types of rain collectors than were found by both air and rain collectors. (B) OTUs of Ascomycota and Basidiomycota detected at different locations. More Ascomycota OTUs were shared by all three locations than those of Basidiomycota. AG, Agassiz, BC; HA, Harrington, PEI; SC, Sainte-Clotilde, QC.

**FIG 2:**
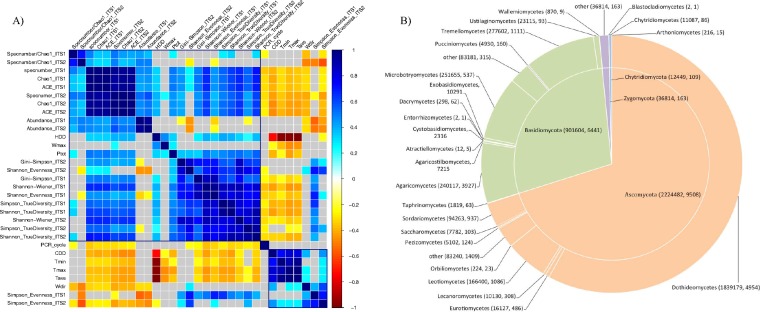
(A) Correlation analysis between mycobiota diversity indices and the climatological parameters. Total precipitation, heating degree days, and the maximum wind speed were positively correlated with fungal abundance and richness indices but negatively correlated with diversity evenness. At a significance level of 0.05, the positive correlations are filled in blue, and the negative correlations are filled in yellow. Wind direction, minimum, maximum and average daily temperatures, cooling degree days, and the number of PCR cycles had the opposite impact on diversity indices. (B) Abundance (number of sequences) and richness (number of OTUs) of each phylum and class in aeromycobiota. PCR_cycle, number of PCR cycles; *T*_max_, mean maximum daily temperature; *T*_min_, mean minimum daily temperature; *T*_ave_, mean average daily temperature; HDD, heating degree days; CDD, cooling degree days; *W*_dir_, wind direction; *W*_max_, maximum wind speed; *P*_tot_, total precipitation.

The number of OTUs found and shared at each sampling location and by different types of spore samplers is summarized in Table 1 and Fig. 1. Approximately 22 to 84% of the total richness represented by Chao1 index (24) was recovered for each sample (Table 1). In general, abundance is associated with higher richness but lower evenness and true diversity, indicating the discovery of rare taxa with increased sequencing depth (Fig. 2A). Based on repeated measures of analysis of variance (ANOVA) and Tukey's honestly significant differences (HSD) *post hoc* tests, the total richness was higher at the beginning (sampling weeks 1 and 5) and the end (sampling week 17) of summer (June to September) but lower during the middle of the summer (sampling weeks 9 and 13) (see Fig. S3 in the supplemental material).

**TABLE 1:** Expected richness (Chao1 index) of total aeromycobiota and air-/rain-borne plant pathogens in identified phyla

Group	Phylum	Relative amt of ITS1 vs ITS2 OTUs	Region	Chao1 index by sampler site and type (mean ± SE)^*a*^								
				Agassiz (*n* = 30)			Harrington (*n* = 34)			Sainte-Clotilde (*n* = 33)		
				BK (*n* = 13)	JB (*n* = 10)	YE (*n* = 7)	BK (*n* = 14)	JB (*n* = 11)	YE (*n* = 9)	BK (*n* = 14)	JB (*n* = 11)	YE (*n* = 8)
Overall	Overall	ITS1 < ITS2***	ITS1	971 ± 78	939 ± 179	1,110 ± 181	888 ± 104	783 ± 119	935 ± 143	1,023 ± 132	969 ± 126	1,015 ± 188
			ITS2	1,029 ± 89	954 ± 178	1,101 ± 97	974 ± 122	837 ± 110	978 ± 167	1,062 ± 127	961 ± 133	1,051 ± 180
	Ascomycota	ITS1 < ITS2***	ITS1	492 ± 32	601 ± 120	583 ± 110	480 ± 74	536 ± 80	541 ± 86	608 ± 84	600 ± 69	502 ± 93
			ITS2	615 ± 48	645 ± 118	623 ± 64	582 ± 87	606 ± 79	589 ± 113	673 ± 80	604 ± 76	566 ± 99
	Basidiomycota	ITS1 > ITS2*	ITS1	451 ± 55	300 ± 64	497 ± 88	389 ± 49	222 ± 42	366 ± 64	376 ± 60	336 ± 70	488 ± 113
			ITS2	400 ± 51	288 ± 64	462 ± 63	381 ± 51	206 ± 34	372 ± 70	377 ± 56	337 ± 76	461 ± 106
	Chytridiomycota	ITS1 > ITS2*	ITS1	1 ± 1	5 ± 2	5 ± 3*	0 ± 0	2 ± 2	2 ± 2*	2 ± 1	3 ± 1	6 ± 2*
			ITS2	1 ± 1	4 ± 2	2 ± 1	1 ± 1	1 ± 1	2 ± 1	1 ± 1	3 ± 2	4 ± 2
	Zygomycota	ITS1 > ITS2***	ITS1	5 ± 2	17 ± 6	6 ± 3	1 ± 1	5 ± 2	6 ± 3	2 ± 1	15 ± 5	7 ± 4**
			ITS2	4 ± 1	12 ± 5	5 ± 2	2 ± 1	3 ± 1	4 ± 2	1 ± 1	9 ± 3	6 ± 4
	Other	ITS1 > ITS2***	ITS1	20 ± 3	17 ± 5	16 ± 6	19 ± 3	23 ± 4	20 ± 5	36 ± 6	26 ± 7	21 ± 7
			ITS2	8 ± 2	10 ± 3	11 ± 4	10 ± 2	17 ± 6	11 ± 5	10 ± 2	8 ± 2	15 ± 7
Pathogen	Overall	ITS1 > ITS2***	ITS1	198 ± 15	178 ± 27	174 ± 39	155 ± 16	181 ± 26	187 ± 26	208 ± 29	179 ± 19	183 ± 32
			ITS2	179 ± 12	141 ± 23	134 ± 20	137 ± 11	146 ± 19	151 ± 26	182 ± 23	151 ± 20	156 ± 28
	Ascomycota	ITS1 > ITS2***	ITS1	164 ± 12	155 ± 24	135 ± 32	128 ± 14	153 ± 22	144 ± 19	174 ± 25	154 ± 15	125 ± 24
			ITS2	141 ± 9	123 ± 20	104 ± 18	110 ± 8	133 ± 17	124 ± 22	156 ± 20	127 ± 17	120 ± 21
	Basidiomycota	ITS1 > ITS2***	ITS1	35 ± 5	18 ± 4	36 ± 8*	30 ± 6	25 ± 7	42 ± 9	32 ± 6	28 ± 6	57 ± 11
			ITS2	39 ± 7	14 ± 4	24 ± 5*	27 ± 5	13 ± 3	23 ± 6	23 ± 4	21 ± 5	32 ± 8

a The Chao1 indices recovered by ITS1 and ITS2 in each phylum were subjected to a Wilcoxon signed-rank test. The Chao1 indices in spore samplers at each sampling location were subjected to a Kruskal-Wallis test. Statistical significance is indicated as follows: ***, *P* ≤ 0.001; **, *P* ≤ 0.01; *, *P* ≤ 0.05.

Only 23% of the total OTUs were found at both the eastern and western coasts of Canada, with more OTUs being recovered at both Harrington and Saint-Clotilde (east). A higher proportion of fungal OTUs were detected by both kinds of rain samplers (a passive wet- and dry-deposition sampler designed by J. Johnson and C. Barnes [JB] and a wet-deposition sampler from Yankee Environmental Systems [YE]) than by both YE and a continuous volumetric sampler from Burkard Manufacturing Co., Ltd. (BK) (Fig. 1A). The core mycota, defined as OTUs observed in >70% of samples from each sampling location, consisted of 23 ITS1 OTUs (0.27%) and 45 ITS2 OTUs (0.56%) but contained almost 44% of the total sequences. Only one Cladosporium OTU (ITS1_OTU 470), containing 1.1% of total sequences, was found in all samples.

Multiresponse permutation procedure (MRPP) analysis showed significant (MRPP *P* < 0.001) differences in the aeromycobiota communities at different sites (MRPP *A* = 0.043, *T* = −23.32) or sampled with different sampler types (MRPP *A* = 0.031, *T* = −17.32), which suggests a low within-group similarity (effect size *A* < 0.1) and high among-group differences (large negative value of *T*). Tukey's HSD tests further suggested homogeneity of dispersions among sampling locations or sampler types (*P* > 0.05), except for BK and YE (*P* = 0.0017), with higher beta-diversity among YE samples.

### Taxonomic composition of air- and rain-borne fungal communities.

Approximately 97% of the total sequences were assigned to four phyla, 90% were assigned to 24 classes, 86% were assigned to 100 orders, 54% were assigned to 242 families, and 61% were assigned to 841 genera. The majority of sequences were classified as Ascomycota (ca. 68.1%) and Basidiomycota (ca. 27.6%) (Fig. 2B). The remaining sequences were assigned to Zygomycota sensu lato (mostly Mucoromycota in the strict sense; 1.13%) and Chytridiomycota (0.38%). The most abundant Ascomycota classes were Dothideomycetes (82.7% of Ascomycota sequences), Leotiomycetes (7.5%), and Sordariomycete*s* (4.2%), while the most observed basidiomycetous classes were Tremellomycetes (30.8% of Basidiomycota sequences), Microbotryomycetes (27.9%), and Agaricomycetes (26.6%) (Fig. 2B). More Ascomycota OTUs were found at all three cities than Basidiomycota OTUs (Fig. 1B). The relative abundance and richness of Ascomycota were significantly higher in samples collected in BK and JB than in YE (p < = 0.01), but their evenness and true diversities were higher in YE and JB, while those of Basidiomycota were significantly higher in samples collected in YE than in BK and JB (Fig. S4).

**FIG 3:**
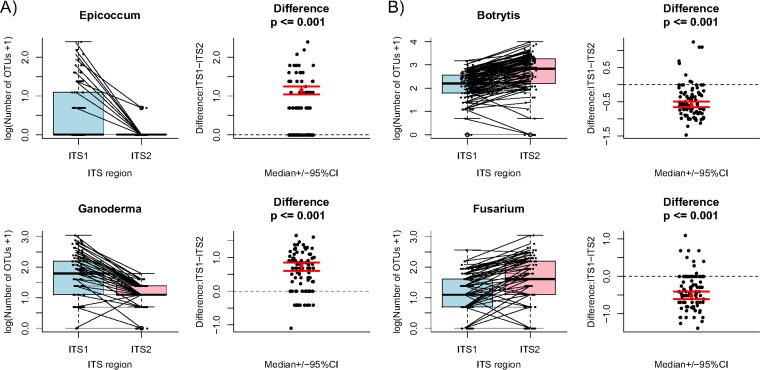
Richness of four fungal genera recovered by ITS1 and ITS2 regions. (A) Epicoccum and Ganoderma. (B) Botrytis and Fusarium. CI, confidence interval.

Interestingly, the fungal richness recovered by ITS1 and ITS2 was significantly different for many fungal groups (Fig. 3; also see Fig. S6). For example, the average number of OTUs and relative abundance recovered by the ITS1 region were higher for Epicoccum and Ganoderma (Fig. 3A) but lower for Botrytis and Fusarium (Fig. 3B) than those recovered by the ITS2 region.

### The shift of composition in aeromycobiota.

**FIG 4:**
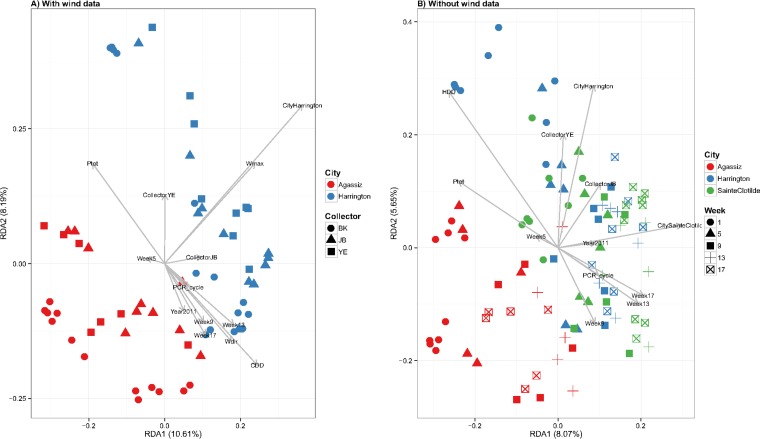
The redundancy analysis (RDA) plots based on the Hellinger distance of the OTU abundance matrices with (A) and without (B) wind data. Aeromycobiota were differentiated in samples collected from the east and west coasts of Canada (A and B), in samples collected from air and rain (A and B), and in samples collected at the beginning of the summer and those collected at the end of the summer (B). The two axes in plot A explained ca. 20% variance, while those in plot B explained ca. 14%. The colors represent different locations, and the shapes represent different types of spore samplers in panel A and different sampling weeks in panel B.

Among all environmental conditions analyzed (Table S1 and Fig. S1 and S2), heating degree day (HDD) and wind direction to the north (*W*_dir_) were positively correlated with daily average, minimum, and maximum temperatures (*T*_ave_, *T*_min_, and *T*_max_, respectively) but negatively correlated with cooling degree day (CDD), total precipitation (*P*_tot_), and maximum wind speed (*W*_max_) (Fig. 2A; see also Fig. S2). Based on the redundancy analysis (RDA), all independent variables collectively explained 21.71% of the total variance of compositional heterogeneity, defined as the total explainable variance (EV), when wind data were included in the model (excluding samples collected from Saint-Clotilde). Collector type explained 21.4% of the EV while the sampling location explained 12.2%. Among other independent variables, 8.2% of EV was explained by sampling year, 11.5% by *T*_max_, 8.7% by *W*_max_, 7.4% by *W*_dir_, and 6.9% by *P*_tot_. When wind data were excluded in the analysis (including all samples), 22.32% of EV was explained. Collector type, the sampling location, and the sampling week each explained 20% of EV. Climate variables (*P*_tot_, HDD, and CDD) collectively explained 13% of EV, and PCR cycles (PCR_cycles) explained 4.6% of EV. The joint effect of all variables resulting from colinearity was 11.5% with wind data and 11.6% without wind data. The ordination plots (ordiplots) (Fig. 4A) showed that aeromycobiota communities differed in compositional structure for samples from each coastal region. Wind speed had a higher impact on samples from Harrington than from Agassiz, while wind direction affected the air samplers more than the rain samplers. Precipitation and temperature had opposite effects on fungal community composition. A seasonal pattern of fungal community composition was also observed, with samples collected at the beginning and end of the sampling season, i.e., end of the spring and beginning of the fall, being separated (Fig. 4B).

### Fungal pathogens.

A total of 4,519 ITS1 and 4,001 ITS2 OTUs were annotated by FUNGuild (25) to 61 and 59 ecological guilds, respectively, of which, 1,248 ITS1 OTUs (representing 158 genera) and 1,031 ITS2 OTUs (representing 163 genera) are considered possible, probable, or highly probable plant pathogens (178 in total). It should be noted that some of these genera are not agricultural pathogens, as shown in Table 2. Of these 178 fungal pathogenic genera, 143 were discovered by both ITS1 and ITS2, while 15 (e.g., Thekopsora and Dothistroma) by were discovered by ITS1 and 20 (e.g., Trichoderma and Magnaporthe) were discovered by ITS2 only. The most abundant genera in this list include Cladosporium (14.5%, including sequences identified as Davidiella and uncultured Cladosporium) (see Table S2 in the supplemental material), Alternaria (4.7%, including Lewia), Ganoderma (2.18%), Fusarium (2.1%, including Gibberella), Ustilago (0.64%, including Pseudozyma), Bipolaris (0.62%, including Cochliobolus), Ascochyta (0.41%), Periconia (0.19%), and Ramularia (0.12%, replaces Mycosphaerella and refers to a broader concept of the genus now prevalent [26]). In addition to Alternaria and Fusarium, other genera containing mycotoxin-producing fungi that were recovered included Aspergillus (0.087%, including Eurotium, Emericella, and several other synonymous genera), Claviceps (0.008%), Penicillium (0.094%, including Eupenicillium but excluding Talaromyces), and Stachybotrys (0.0006%). The relative abundances of selected genera collected by different samplers during the growing seasons are shown in Fig. S5.

**TABLE 2:** The impacts of the types of spore samplers and other environmental variables on the recovery of selected pathogenic fungal genera determined by negative binomial generalized linear models using a rarefied community abundance matrix^*a*^

Genus	Best collector	Description	Total abundance	glm.nb-selected model	Collector		City		Week		Year		Total precipitation		Maximum daily temp	
					Coeff	*q* value	Coeff	*q* value	Coeff	*q* value	Coeff	*q* value	Coeff	*q* value	Coeff	*q* value
Aspergillus	BK	Crop pathogen, saprobe	126	Collector	BK, JB, YE	0.024362										
Chalastospora	BK	Crop pathogen, saprobe	16	Collector+wk	BK, YE, JB	**0.000004**			13, 9, 1, 5, 17	0.016073						
Cladosporium	BK	Crop pathogen, saprobe	18,493	Collector+yr	BK, JB, YE	**0**					2010, 2011	0.048471				
Drechslera	BK	Crop pathogen	64	Collector+*T*_max_	BK, JB, YE	0.00181									**0.8932**	0.024785
Entyloma	BK	Smut	37	Collector+yr	BK, YE, JB	**0**					2010, 2011	0.032209				
Mycocentrospora	BK	Crop pathogen	25	Wk+*T*_max_+collector	BK, JB, YE	0.033851			1, 5, 9 = 13 = 17	**0**					**3.1496**	**0**
Phellinus	BK	Tree pathogen	172	*T*_max_+collector+city	BK, YE, JB	**0.000201**	AG, SC, HA	0.002018							−1.0162	**0**
Piptoporus	BK	Tree pathogen	162	Collector+city	BK, YE, JB	**0.000005**	SC, AG, HA	0.001093								
Pleospora	BK	Crop pathogen, saprobe	457	Collector+*T*_max_+wk+city+yr	BK, JB, YE	**0**	AG, HA, SC	0.007305	17, 13, 1, 9, 5	0.002264	2010, 2011	0.024932			**1.5221**	**0.000133**
Torula	BK	Crop pathogen, saprobe	34	Collector	BK, JB, YE	0.007757										
Aureobasidium	JB	Crop pathogen, saprobe	9,358	Collector+yr+*T*_max_	JB, YE, BK	**0**					**2011, 2010**	**0.00013**			−0.4144	**0.002063**
Devriesia	JB	Crop pathogen, saprobe	64	Collector	JB, BK, YE	0.047946										
Knufia	JB	Tree pathogen, saprobe	16	Collector	JB, BK, YE	0.008706										
Microdochium	JB	Crop pathogen	83	Collector	JB, YE, BK	0.003572										
Phoma	JB	Crop pathogen, saprobe	222	City+yr+collector	JB, YE, BK	0.01586	HA, AG, SC	**0**			**2011, 2010**	0.00876				
Rhynchosporium	JB	Crop pathogen	36	Wk+*T*_max_+collector	JB, BK, YE	0.041763			1, 13, 5, 9, 17	**0**					−0.919	0.00533
Stagonospora	JB	Crop pathogen	18	City+wk+collector	JB, BK, YE	0.016794	SC, HA, AG	0.00177	1, 9, 5, 13, 17	0.002264						
Fusarium	YE	Crop pathogen	2,412	Yr+collector+city	YE, JB, BK	0.011605	SC, HA, AG	0.01266			2010, 2011	**0.00084**				
Ganoderma	YE	Tree pathogen	2,884	Wk+collector+city	YE, JB, BK	**0.000038**	SC, AG, HA	**0.000213**	9, 13, 17, 5, 1	**0**						
Itersonilia	YE	Crop pathogen	120	*T*_max_+collector+wk	YE, JB, BK	**0.00034**			5, 1, 13, 17, 9	0.002865					−1.339	**0**
Leptosphaeria	YE	Crop pathogen	56	Collector+*P*_tot_+*T*_max_	YE, JB, BK	**0.000228**							**0.8045**	0.049099	**0.7442**	0.024785
Phaeomoniella	YE	Pathogen of woody plants	13	Collector	YE, JB, BK	0.009369										
Podosphaera	YE	Powdery mildew	26	Collector	YE, JB, BK	0.01106										
Powellomyces	YE	Chytrid	270	Collector+city+yr+wk	YE, JB, BK	**0**	SC, AG, HA	0.009114	13, 5, 9, 1, 17	0.00642	**2011, 2010**	0.017988				
Rhizosphaera	YE	Conifer endophyte	18	Collector+city+*T*_max_	YE, JB, BK	**0.000228**	SC, HA, AG	0.02058							−0.844	0.04434
Taphrina	YE	Tree pathogen	18	Wk+collector	YE, JB, BK	0.012235			1, 5, 13, 9, 17	**0.000516**						
Ustilago	YE	Smut	999	Collector	YE, JB, BK	**0**										
Ascochyta		Crop pathogen	761	City+*T*_max_			AG, SC, HA	**0**							**0.5771**	0.006117
Camarosporium		Crop pathogen, saprobe	45	Wk+city			AG, HA, SC	0.021776	5, 1, 9, 13, 17	**0.000003**						
Erysiphe		Powdery mildew	16	City			AG, HA, SC	0.00669								
Melampsora		Rust	92	City+wk			AG, SC, HA	0.00261	1, 17, 13, 5, 9	0.043557						
Ramularia		Crop pathogen	184	City+wk+*T*_max_			AG, HA, SC	**0.000187**	1, 5, 17, 9, 13	0.005583					**0.9692**	0.010631
Sawadaea		Powdery mildew	154	City+wk+yr+*P*_tot_			AG, SC, HA	**0**	5, 9, 13, 1, 17	**0.000004**	2010, 2011	0.036473	**1.1831**	0.045443		
Urocystis		Smut	65	Wk+city+*T*_max_			AG, SC, HA	**0**	1, 5, 3, 17, 13	**0**					**1.3927**	0.002063
Alternaria		Crop pathogen	7,349	City+wk+*P*_tot_+yr			HA, SC, AG	**0**	17, 13, 9, 1, 5	**0.000003**	2010, 2011	0.036473	−0.3736	0.00525		
Athelia		Crop pathogen	26	*T*_max_+city			HA, SC, AG	**0.000663**							−1.6243	0.00533
Bipolaris		Crop pathogen	1,423	City+*T*_max_+yr+wk			HA, SC, AG	**0**	13, 17, 9, 1, 5	0.002264	2010, 2011	0.01667			**1.2434**	**0**
Blumeria		Powdery mildew	294	City			HA, SC, AG	0.017609								
Mollisia		Root endophyte	25	City+*P*_tot_			HA, SC, AG	**0.00004**					**0.7922**	0.003568		
Phaeoacremonium		Pathogen of woody plants	17	Yr+city+*P*_tot_+*T*_max_			HA, SC, AG	0.005822			**2011, 2010**	**0.000053**	−5.4343	0.049099	**2.7788**	0.017662
Ramichloridium		Crop pathogen, saprobe	11	City			HA, AG, SC	0.021776								
Mycena		Wood saprotroph, probable pathogen	32	*T*_max_+city+yr			SC, AG, HA	**0.000004**			**2011, 2010**	0.048471			−5.1047	0.011637
Periconia		Crop pathogen	324	City+wk+yr			SC, AG, HA	**0.00002**	5, 13, 17, 1, 9	**0.00001**	**2011, 2010**	0.017988				
Pestalotiopsis		Crop pathogen	26	City			SC, AG, HA	**0.000978**								
Streptobotrys		Crop pathogen	18	City			SC, AG, HA	0.00599								
Typhula		Saprobe	12	*T*_max_+city			SC, AG, HA	0.043383							−2.2546	0.010631
Hymenula		Crop pathogen	16	Yr							**2011, 2010**	**0.000015**				
Gnomonia		Crop pathogen	67	Wk					1, 5, 9 = 13 = 17	**0**						
Pucciniastrum		Rust	12	Wk					5 = 13 = 9 = 17, 1	0.015782						
Ophiognomonia		Tree pathogen	29	Wk+*P*_tot_					1, 9, 5, 17, 13	**0**			**12.1449**	0.009656		
Strelitziana		Crop pathogen	22	*P*_tot_+*T*_max_									−21.1343	**0**	**0.8991**	0.002063
Puccinia		Rust	62	*P*_tot_+yr							2010, 2011	0.017988	−1.4503	0.003568		

a The impacts of the categorical predictor variables, including spore samplers, cities, sampling weeks, and years, are represented in the form of sequentially arranged levels in order of the coefficients of respective levels. The impacts of the numeric variables, including total precipitation and maximum daily temperature, are represented by the standardized coefficients, with positive ones in bold. The *q* values are adjusted from *P* values generated by likelihood ratio statistics using the false discovery rate approach. Statistically significant *q* values are in bold and set at 0.05/50 = 0.001. The genera are sorted first by the performance of spore samplers, then by their recovery at each city. The type of spore sampler showed impact only on the first 27 genera in this table. The spore sampler type that had the highest efficiency in recovering a fungal genus was selected as the best collector for that taxon. Sample size, 1,532 sequences.

**FIG 5:**
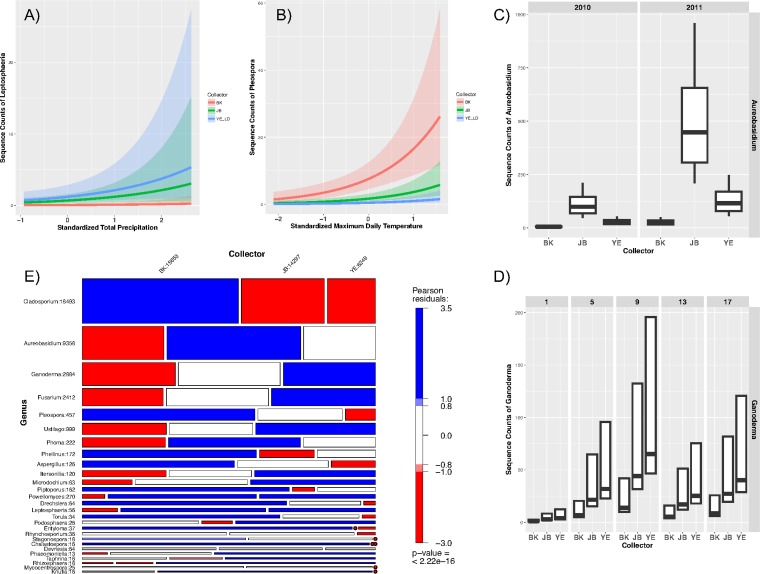
(A to D) Abundance of Leptosphaeria (A), Pleospora (B), Aureobasidium (C), and Ganoderma (D) recovered by three types of spore samplers and under different environmental conditions based on a negative binomial generalized linear regression model, summarized in Table 2. (E) Mosaic plots using HSV shading with user-defined cutoffs (0.8 and 1) based on the Pearson residuals of an independence model for rarefied community (sample size, 1,532), which was then double square root-transformed. The width and height of tiles represent the relative frequencies of spore samplers and genera. The area of each tile is proportional to the observed frequencies of the associated genus recovered by each sampler, with the left tile for BK, the middle tile for JB, and the right tile for YE in each row. The color of each tile represents the level of the residual, with blue, red, or white indicating that the level is more than, less than, or similar to observations under the null hypothesis for a test of independence. The colored tiles contribute to the significance of the chi-square test result. The genera are ordered by abundance, which is shown by the number following each generic name. The numbers following spore samplers represent total abundance of selected pathogens shown on the figure.

**FIG 6:**
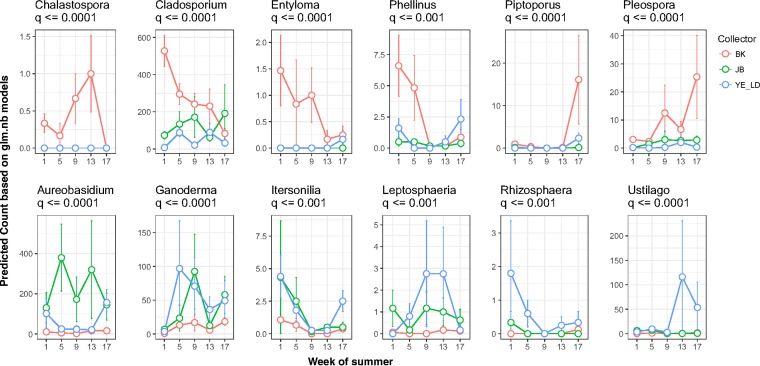
The glm.nb-predicted abundance of 12 phytopathogenic fungal genera that are preferably (*P* < = 0.001) collected by air (top panel) or rain (bottom panel) samplers during the growing seasons. The error bars show the standard errors of the average abundance of a genus at a sampling week across all three cities and over the two growing seasons.

The negative binomial generalized linear regression (glm.nb) model with the log link function was used to assess the impacts of independent variables based on rarefied generic abundance matrices of both ITS1 and ITS2 sequence counts (sample size, 1,532). The result is summarized in Table 2 for 52 genera, with 38 in Ascomycota, 13 in Basidiomycota, and 1 in Chytridiomycota. Negative binomial modeling suggests that the sampler types differ in their abilities to collect members of 27 fungal plant (mainly crop) pathogens as follows: Piptoporus (wood rotting tree pathogen), Pleospora, Chalastospora, Cladosporium, Entyloma, Phellinus (wood rotting tree pathogen), Torula, Aspergillus, Drechslera (including Pyrenophora and Setosphaeria), and Mycocentrospora were collected more by BK; Knufia, Microdochium (including Monographella [27]), Devriesia, Aureobasidium, Stagonospora, Phoma, and Rhynchosporium were collected more by JB; and Podosphaera, Phaeomoniella, Ustilago (including Pseudozyma), Rhizosphaera (conifer endophyte and leaf pathogen), Powellomyces, Leptosphaeria, Itersonilia, Ganoderma (wood rotting tree pathogen in Canada and crop pathogen in the tropics), Taphrina (tree pathogen), and Fusarium were collected more by YE (Table 2, upper portion). The glm.nb model also assessed the geographic and temporal heterogeneity of pathogen distribution under the impact of weather conditions (total precipitation and maximum daily temperature), as shown in Table 2 and Fig. 5A to D. The recovery efficiencies of samplers for the 27 genera listed in the upper part of Table 2 are summarized by a mosaic plot (Fig. 5E). The width, height, and area of each tile represent the proportion of the observed frequencies that a genus was collected by a spore sampler. A chi-square test of independence showed that spore sampler types had significant impact on the recovery of many genera (*P* < 2.2e−16). For each tile, blue or red, respectively, represents a higher (positive) or lower (negative) level of the residuals (observations) than expected while white indicates a very small level of the residuals. Figure 6 shows the abundance predicted by glm.nb of 12 phytopathogenic fungal genera that are preferably (*P* < = 0.001) collected by air (top panel) or rain (bottom panel) samplers through the growing season.

The types of spore samplers did not significantly impact the recovery of the fungal plant pathogens listed in the lower part of Table 2. These genera, however, were recovered differently at each sampling location or sampling time or under specific weather conditions. For example, both Bipolaris and Alternaria were recovered more at the end of the summer in the east coast cities, while Ascochyta was more prevalent in Agassiz (west coast).

## DISCUSSION

Few studies have characterized the compositional structure and diversity of aeromycobiota (11, 28–34). As a major source of biological aerosols, fungal spores consist of 4 to 45% of air particulate matter with spatial and temporal distribution patterns, which can be attributed to environmental filtering, fungal life cycles, and dispersal limitations imposed by spore size (28, 30, 35–37). The spatial-temporal compositional structure of the aeromycobiota of Canada, one of the largest agricultural producers in the world, has not been well investigated. In this study, we characterized the aeromycobiota at selected sites in Canada and evaluated the performance of three types of spore samplers in collecting air- and rain-borne fungi, especially plant pathogens.

Overall, Dothideomycetes and Agaricomycetes were among the dominant classes, and a much smaller number of taxa were recovered from basal fungal lineages (e.g., Chytridiomycota), which correlates with the relative ITS amplification success rates for these taxa using standard ITS fungal primers observed by Schoch et al. (38). Other biases in diversity assessments using ITS markers may be attributed to factors such as the presence of multicopy orthologs, a lack of discrimination among cryptic taxa, the 97% identity threshold for OTU clustering, sequencing error rates, or low taxonomic coverage in reference databases, as has been evaluated and reviewed in detail previously (39–43). Our results also reflect different discriminating powers of the ITS1 and ITS2 regions or difficulties in amplifying particular taxonomic groups using ITS5/4 primers (Fig. 3). Targeted bio-surveillance of fungal pathogens using a metabarcoding approach, thus, should select the ITS region and appropriate primer sets to maximize recovery.

A higher prevalence of Ascomycota was observed in samples collected by BK and JB collectors (p < = 0.001) but not by the active rain samplers (YE) (Fig. S4). In addition, among the 16 samples where the Basidiomycota proportion was >50%, 11 were collected by active rain samplers. Such observations may reflect a collecting bias of the samplers or indicate a higher concentration of basidiospores in the rain and a higher abundance of ascospores in the air. Despite the high abundance of Ascomycota, their richness was only significantly higher than that of Basidiomycota in JB samples. One possible explanation is that JB samplers were open to collect both air and rain deposition; as spores of Basidiomycota are consistently smaller than the spores of Ascomycota (9, 44), the former thus have a better chance to be deposited and captured by JB with or without rainfall. In contrast, the filamentous growth forms of Ascomycota are smaller than those of Basidiomycota (9, 36, 45) and more easily aerosolized for long-distance dissemination. This is partially supported by the fact that the number of Ascomycota OTUs found at all three locations was approximately double that of Basidiomycota, indicating a lack of long-distance dispersal capacity for many Basidiomycota. Furthermore, air samplers performed better in capturing rare Ascomycota taxa than rain samplers, as represented by the ratio between Ascomycota and Basidiomycota evenness indices (<1.0), indicating a relatively more uniform Basidiomycota community in the air (see Fig. S4 in the supplemental material).

The wide range of diversity indices and extremely low evenness, together with the fact that very few OTUs were shared by all samples, suggested a highly diversified air- and rain-borne fungal community structure over the seasons and locations. It was unsurprising that a regional distribution pattern of air- and rain-borne fungi was observed on the east and the west coasts of Canada (Fig. 1 and 4). Interestingly, however, among the Ascomycota OTUs shared by Agassiz and the two eastern sampling locations, a significantly higher proportion was detected at both Agassiz and Harrington (both coastal locations) than at both Agassiz and Saint-Clotilde (inland locations), suggesting a distinct mycobiota composition in the terrestrial versus oceanic air. Fröhlich-Nowoisky et al. (34) observed an increased ratio of airborne Ascomycota versus Basidiomycota species richness when the distance from the land to the ocean was increased, which was not revealed in our data. Such disparities may reflect different methodologies used to process and analyze the environmental samples. For instance, Fröhlich-Nowoisky et al. (34) grouped the clones of ITS amplicons based on restriction fragment length polymorphism (RFLP) results and then sequenced a representative clone for each RFLP type. The proportion of fungal species revealed by this approach may be lower than that resulting from a metabarcoding approach. Indeed, the observed richness in the current study was similar to or slightly higher than that of previous studies (28, 29, 46).

In addition, at all three sampling locations, the relative abundance of Ascomycota sequences (>64%) was significantly higher than that of Basidiomycota sequences (<30%). Similar aeromycobiota composition was also observed in China during hazy days (29), in some regions in North America (36), and in marine air (34); the opposite pattern, i.e., higher richness and abundance in Basidiomycota than Ascomycota, however, was observed in the air of central Europe and over the Amazon rainforest (9, 28). These DNA-based studies reflected the total aeromycobiota compositional structure, including both viable and metabolically inactive spores and fragments of fungi, whereas the active aeromycobiota is dominated by Ascomycota, based on transcriptome sequencing (RNA-seq) data (9). The spatial distribution pattern and structural differences in total and active aeromycobiota suggested that both the spores and vegetative growth forms of Ascomycota are small enough to be aerosolized, while Basidiomycota may disseminate primarily in a resting spore form with minimal metabolic activity while dispersed by air (9). Studies also showed that the composition of aeromycobiota at a regional scale was correlated to, but distinctly different from, that of the local phyllosphere and soil, with a majority of community members attributed to distant sources (9, 37).

Although rain samplers collected more Basidiomycota and air samplers collected more Ascomycota, plant pathogens in these two most abundant phyla did not consistently follow the same pattern (Table 2). For example, Piptoporus, Entyloma, and Phellinus in Basidiomycota were recovered more in BK. The majority of the fungal spores, such as rust fungi (e.g., Puccinia), Alternaria, Drechslera, and Cladosporium (16, 18), depend on wind for dispersal. These fungi either were recovered more by air samplers (Cladosporium and Drechslera), or their abundance decreased in wet weather (Puccinia and Alternaria). However, smut fungi (e.g., Ustilago) and powdery mildews (e.g., Podosphaera) are also considered wind-disseminated fungi; they were collected more by rain samplers in the current study. Spores of these pathogens disseminate through wind from distant sources, but precipitation facilitates their deposition from the air (47). For spores or fragments from local sources, e.g., chasmothecia of powdery mildews on woody deciduous perennial hosts such as grapevine and lilac, water availability promotes spore production and liberation (48). These spores are dispersed by rain from infected host tissues, e.g., leaves or stems, to other substrates (49). Hydrophobins in cell walls of filamentous fungi also play an important role in surface attachment and water-mediated dispersal (50, 51). We recovered significantly more Fusarium spp. and Phoma spp. in rain samples, possibly a consequence of splash dispersal of their sticky conidia (14, 52). Some species in Fusarium, e.g., Fusarium avenaceum and Fusarium acuminatum, are highly effective ice-nucleating bio-aerosols, i.e., able to initiate ice formation at warmer temperatures (11, 51, 53), and may contribute to cloud formation and deposit through precipitation. These biological ice nuclei, including spores of rusts (e.g., Puccinia spp.) and kernel bunt (e.g., Tilletia spp.), can reach high altitudes for long-distance dissemination (54). Our findings agree with those of Kivlin et al. (37) that the dispersal of fungal spores is both a random and deterministic process. Therefore, sampling a wider vertical air column and using different collecting methods would facilitate the capture of a higher diversity of economically important fungal pathogens.

Our data further indicated that the air- and rain-borne fungal communities varied in species composition over time during the sampling season, in contrast to some culture-based studies which concluded that microfungal communities are relatively stable over a long period (47, 55). There was a clear delineation in the composition of fungal species through the sampling season each year (Fig. S3), with the fungal community usually less diverse in the middle of the summer (July) than at the beginning (end of spring) and the end (beginning of autumn) of the growing season. The temporal heterogeneity in the fungal communities was also observed by Nicolaisen et al. (56) in northwestern Europe and was often attributed to nutritional demands and survival strategies. Surrounding vegetation is particularly important for the life cycles of some plant-pathogenic fungi that are associated with particular crop or plant growth stages (57). In temperate climates, fungal species sporulate seasonally, with a flush of sexual spores of many pathogenic microfungi, such as Sclerotinia sclerotiorum and Claviceps purpurea, occurring in spring when host crops are flowering and then a Basidiomycota spore flush in the autumn when winter crops emerge (58). The long- or short-distance dispersal of spores in the air or rain can be further affected by climatological conditions for growth and transportation (59). In this project, climatological parameters differed in the amount of variance they explained for air- and rain-borne fungal abundance and richness. The total precipitation (*P*_tot_) and wind direction to the north (*W*_dir_) had opposite effects on mycobiota diversity in comparison with those of the temperature and the maximum wind speed (*W*_max_), suggesting that a hot and dry summer may be unfavorable for the growth and distribution of fungi in general (Fig. 1B) (60, 61). Talley et al. (62), however, stated that high humidity is essential for growth of most mesophilic fungi and was considered a good predictor of fungal prevalence. The total variance explained by the RDA model (ca. 22%) was higher than that of Grinn-Gofroń and Bosiacka (63) (ca. 16.5%) and suggested that mean air temperature was the most important factor affecting the composition of airborne fungal spores. Overall, our results suggest that the northeast-bound winds that prevail on the North American continent during the spring and summer contribute significantly to the fungal richness and to the prevalence of fungal pathogens in Canada's air.

We summarize the collecting preference of samplers, the spatial and temporal distribution patterns, and the influence of climate conditions on the detection of 52 plant fungal pathogens in Table 2 and Fig. 5, which may guide the selection of appropriate spore samplers for crop pathogen collection. For example, Pleospora spp. were collected at significantly higher levels by BK at Agassiz (west), while Phoma spp. were recovered more by JB and YE at Harrington (east). Ustilago spp. and Leptosphaeria spp. were all recovered more by rain samplers (YE and JB); however, they did not show geographic distribution preference. Selecting the appropriate types of samplers would, therefore, increase the recovery rate of these targets. On the other hand, the type of spore sampler did not show collecting bias for genera such as Melampsora spp., Urocystis spp., Alternaria spp., and Bipolaris spp., which, however, were more abundant in either the west or the east coastal regions. Interestingly, for the powdery mildews, Erysiphe spp. were more prevalent at Agassiz, but Blumeria spp. were more abundant on the east coast, possibly a consequence of the distribution of suitable hosts.

### Conclusions.

We performed an exhaustive characterization of the aeromycobiota of agricultural experimental stations on the east and west coasts of Canada and one interior site. The efficiency and collection preference of spore samplers in recovering fungal pathogens from air and rain masses were addressed, providing guidelines for selecting the optimal samplers for certain taxonomic groups. The capture and analysis of plant pathogen inocula dispersed in air and rain are powerful tools for assessing disease potential, thus assisting in plant pathology studies, as well as in national biovigilance monitoring and pest management efforts.

## MATERIALS AND METHODS

### Collection of air and rain samples and associated climatological data.

Spore-sampling equipment was used to collect air and rain samples at three agricultural experimental stations operated by Agriculture and Agri-Food Canada (AAFC), located at Agassiz, BC, Sainte-Clotilde, QC, and Harrington, PEI. The three sampling locations in the study have roughly similar latitudes although Agassiz (west) and Harrington (east) are coastal, while Saint-Clotilde (east) is more inland (see Table S1 in the supplemental material). Three different samplers were installed at each site: a continuous volumetric sampler ([BK] the Cyclone sampler for field operation; Burkard Manufacturing Co. Ltd., Rickmansworth, England), an automated wet-deposition sampler ([YE] Model TPC-3000 total precipitation collector; Yankee Environmental Systems, Inc., Turners Falls, MA, USA), and a passive wet- and dry-deposition sampler made in-house ([JB] designed in 2004 by J. Johnson and C. Barnes at USDA-ARS for monitoring Asian soybean rust). All rain samplers were installed on the ground in the middle of a field of growing crops, while BK samplers were installed about four feet above ground. The BK instrument is a non-filter-based system for sampling air-dispersed particulate matter, which is collected directly into a sterile 1.5-ml microcentrifuge tube in the field. Particle size is in the 1-μm range. BK samplers normally collected airborne dry particles but often trapped moisture, and samples were then treated as wet samples. The YE instrument collects wet deposition into a collection bucket only during precipitation events. It is expected to capture spores that are removed from the air by precipitation or by water splash from nearby plants and less likely to capture spores in moving air currents. A moisture sensor triggers the opening and closing of the bucket lid. The collected rainwater was filtered in the lab by vacuum filtration through a 47-mm disposable filter funnel equipped with a 0.45-μm-pore-size cellulose nitrate filter (Whatman catalog no. 1920-1441; Sigma-Aldrich, Oakville, Ontario, Canada) to collect spores and other particulate matter. The JB unit consists of a 17-inch-diameter plastic funnel mounted on a pole, which is open at all times to wet and dry deposition. Rain was filtered directly in the field through a filter assembly unit (same make and filter pore size as used for the YE samples) attached to the base of the funnel. Particulate matter deposited in the funnel as dry deposition was washed down onto the filter during rain events. Occasionally at some sites during weeks with heavy rain or strong wind events, the 0.45-μm-pore-size JB filter became plugged, and the extra water in the filter unit was brought to the lab to be filtered and combined with the rest of the sample.

Collectors were installed for 17 weeks from June to September in 2010 and 2011, i.e., from June 1 (initial setup) to September 28 (last collection day) in 2010 and from May 31 to September 27 in 2011. Samples were collected after 7 days of exposure in the field. Five samples were collected each year (weeks 1, 5, 9, 13, and 17). Some weeks had no precipitation and therefore no YE sample: Agassiz, weeks 5 and 9 in 2010 and week 13 in 2011; Harrington, week 17 in 2011; Saint-Clotilde, week 5 in 2010 and week 1 in 2011. A total of 98 air and rain samples were collected from Agassiz (*n* = 30), Harrington (*n* = 34), and Saint-Clotilde (*n* = 34).

Climatological data, obtained from Environment and Climate Change Canada's meteorological service (http://climate.weather.gc.ca/), included temperature-associated variables (in degrees Celsius) such as average temperature (*T*_ave_), minimum temperature (*T*_min_), maximum temperature (*T*_max_), heating degree day (HDD) and cooling degree day (CDD), wind direction (*W*_dir_, number of degrees from north) and maximum speed (*W*_max_, in kilometers/hour), and total precipitation (*P*_tot_, in millimeters). Cooling degree day is defined as the demand for energy to cool a building and is derived from subtracting a base temperature, e.g., 18°C, from the daily average temperature. The heating degree day is defined as the demand for energy to heat a building and is calculated by subtracting daily average temperature from a base temperature. A summary of metadata for the 98 samples can be found in Table S1 in the supplemental material.

### Sample processing and genomic DNA extraction.

Samples (YE precipitation, JB filter assembly units, and BK microcentrifuge tubes) were collected by collaborators at each location and shipped by overnight courier to the Hambleton lab at AAFC Ottawa for next-day processing. After the YE water was filtered in the lab, the YE and JB filters were removed from the filter assembly units and cut in half. One half was stored as a frozen voucher at −80°C. The other half was accordion folded and placed in a 1.5-ml tube with 800 μl of 10 mM Tris buffer, incubated at 65°C for 5 min, sonicated for 1 min two times to release trapped particles from the filter pores, and centrifuged at 10,000 rpm for 2 min to concentrate the particles. The filter paper was then removed from the tube, and the remaining buffered sample was dried down in a Savant DNA 120 SpeedVac concentrator (Thermo Fisher Scientific, Waltham, MA, USA). DNAs of spiked YE and JB samples with known quantities of Puccinia graminis spores were extracted using different commercial kits, including a PowerSoil kit (Mo Bio Laboratories Inc., Carlsbad, CA, USA) and OmniPrep kit (G-Biosciences, St. Louis, MO, USA). The amount of P. graminis DNA in the extracts was quantified using quantitative PCR (qPCR) (64), which demonstrated that the filter processing and DNA extraction methods were effective for spore recovery (data not shown). Because BK samples were collected directly in Eppendorf tubes, they were processed according to the procedures listed above, excluding the filter removal steps required for the JB/YE samples. Total genomic DNA was extracted from the dried filtrate samples individually using an OmniPrep kit (G-Biosciences, St. Louis, MO, USA) for the BK and YE samples or a PowerSoil kit (Mo Bio Laboratories, Inc., Carlsbad, CA, USA) for the JB samples, which trapped various amounts of soil particles as dry deposition. The result from each sample was a mixed pool of DNA extracted from any species present in the sample (e.g., fungal spores, bacterial cells, pollen, etc.). The extracted DNA was stored at −80°C.

### PCR and 454 pyrosequencing.

Genomic DNA extracted from environmental samples was diluted to 1/10 and 1/100 dilutions to reduce the effects of PCR inhibitors. The universal fungal ITS primers, ITS5 (forward, GGAAGTAAAAGTCGTAACAAGG) and ITS4 (reverse, TCCTCCGCTTATTGATATGC), were fused with a unique 10-nucleotide barcode for multiplexing samples. Products of eight replicate reactions for each PCR library were pooled to limit the effects of PCR bias and increase the detectable biodiversity in each sample. Partial fusion primers were developed to avoid individual adaptor ligations within each amplicon library. These primers combine our standard universal primer sets with unique 10-base multiplex identifier (MID) adaptors (Roche) (Table S1). The PCR and 454 pyrosequencing reactions and conditions were described previously by Chen et al. (65). The 454 pyrosequencing technology was used because other sequencing platforms either were unavailable or when this study began in 2009 could produce only very short sequences.

### Classification and analysis of pyrosequencing data.

The 454 pyrosequencing data were processed using the bioinformatics pipeline described by Chen et al. (65) with the following modifications. (i) Raw sequences of each sample were separated based on the sequencing direction and labeled as forward (sequenced from the ITS5 5′ primer end) or reverse (sequenced from the ITS4 3′ primer end) during the demultiplexing step; reverse sequences were reverse complements of the original orientation. (ii) Quality-trimmed forward and reverse sequences were subjected to chimera checking and then clustered at 97% similarity. Forward sequences contained mainly ITS1 and 5.8S regions and are referred to as ITS1; reverse sequences contained mainly ITS2 and 5.8S regions and are referred to as ITS2. (iii) The OTUs were classified by the Ribosomal Database Project (RDP) classifier (66) using the UNITE (User-friendly Nordic ITS Ectomycorrhiza) fungal ITS database (version 7.0, release date 1 August 2015) as the reference database with a confidence level of 0.8. The forward and reverse sequences formed OTU tables that are referred to as ITS1_OTU and ITS2_OTU, respectively. Some generic names in the taxonomic lineages in the OTU tables were replaced by accepted generic names, including those imposed by the recent adoption of a single-name nomenclature for fungi (67), before downstream analysis was performed (Table S2) (65). For example, Mycosphaerella was replaced by Ramularia (26), and Monographella was replaced by Microdochium (27). Because of the rapid rollout of some of these taxonomic changes, however, some recently accepted synonymies may not have been implemented in the pipeline and are not included in Table S2. Other genera, such as Acremonium, remain phylogenetically unresolved and undoubtedly polyphyletic. Although we have done our best to correct these inconsistencies in our pipelines, the occurrence or cooccurrence of these names in some tables and figures is an unavoidable consequence of the volume of data, the numbers of taxa being processed, and the different taxonomies accepted at the times when some analyses were run. OTUs were classified to functional groups using FUNGuild (25).

All statistical analyses were done in the R environment. Because ITS1_OTU and ITS2_OTU were formed by amplicons from different regions of the ITS barcodes, diversity indices of putative species (OTUs) were calculated for each OTU table separately. R packages vegan (68), BiodiversityR (69), HierDiversity (70, 71), and RAM (72) were used for community diversity analysis. The correlation among diversity indices and numeric independent variables were calculated using the R function cor. To examine how the aeromycobiota diversity changed through the sampling season, we used the lme function in the R package nlme (73) to perform the repeated measures of ANOVA (PMANOVA) analysis, with diversity indices of each type of sampler at each location being compared at five time points (week). The PMANOVA model was further subjected to *post hoc* tests using the glht function in the R package multcomp (74) to examine if diversity was different between sampling weeks. The OTUs were combined for the calculation of a taxonomic abundance matrix (TAM) at each taxonomic rank. OTUs and TAM were Hellinger transformed and then standardized with means equaling to zero, unless otherwise stated. To compare the richness for selected fungal groups at each taxonomic rank recovered by ITS1 and ITS2, we used the R function wilcox.test. To examine the beta-diversity of fungal communities, the function cca in the vegan package was used to perform the redundancy analysis (RDA). The samples from Saint-Clotilde were excluded from RDA when the wind data were included from modeling because wind data were missing for this location in both sampling seasons.

To investigate the impacts of independent variables on the detection of plant pathogens, we used the glm.nb function in the R package MASS (75) for negative binomial linear regression modeling of genus abundance (rarefied to a sample size of 1,532), using the log link function. The categorical variables include spore samplers, locations, and sampling week and year, while the numeric variables include total precipitation and maximum daily temperature, which were standardized to zero mean and unit variance. The *q* values were adjusted from *P* values generated by likelihood ratio statistics using the false discovery rate. Statistically significant *q* values were set at 0.05/50 = 0.001. To visualize the chi-square hypothesis test of independence of sequence counts and categorical variables (e.g., sampler types and geographic location), the mosaic function in the R package vcd (76) was used. Mosaic plots were generated using hue-saturation-value (HSV) shading (77) with user-defined cutoffs (0.8 and 1) based on the Pearson residuals of an independence model for double square root-transformed rarefied community (sample size, 1,532). R packages RAM (72), reshape2 (78), lattice (79), corrplot (80), and ggplot2 (81) were used for manipulating OTU tables and visualization.

### Accession number(s).

All ITS metabarcoding sequences are available through the NCBI Sequence Read Archive (SRA) under study accession number SRP095430 and BioSample accession numbers SRS1872326 to SRS1872422.

## Supplementary Material

Supplemental material
