# Supplementary material for: Assessing Performance of Spore Samplers in Monitoring Aeromycobiota and Fungal Plant Pathogen Diversity in Canada
Source: Appl Environ Microbiol. 2018 Apr 16;84(9):e02601-17. doi: 10.1128/AEM.02601-17 (PMC5930333; doi:10.1128/AEM.02601-17)
Supplement: Supplemental material [file supp_84_9_e02601-17__index.html]

Supplemental material 

# Assessing Performance of Spore Samplers in Monitoring Aeromycobiota and Fungal Plant Pathogen Diversity in Canada

## Supplemental material

- Supplemental file 1 -

  Summary of the samples used in the current study (Table S1); list of synonyms substituted for accepted generic names in the current study (Table S2); sampling locations and average sequence counts (Fig. S1); climatological data of sampling time points (Fig. S2); shift of diversity indices over the sampling weeks (Fig. S3); ratio of diversity indices of Ascomycota and Basidiomycota (Fig. S4); observed relative abundance of selected genera recovered by each spore sampler through the sampling season (Fig. S5); fungal taxa with number of OTUs recovered by ITS1 and ITS2 significantly different at taxonomic levels (Fig. S6).

  PDF, 1.2M
