# Supplementary material for: Assessing Performance of Spore Samplers in Monitoring Aeromycobiota and Fungal Plant Pathogen Diversity in Canada
Source: Appl Environ Microbiol. 2018 Apr 16;84(9):e02601-17. doi: 10.1128/AEM.02601-17 (PMC5930333; doi:10.1128/AEM.02601-17)
Supplement: Supplemental material [file AEM.02601-17_zam009188474s1.pdf]

Supplemental Materials

Tables

Table S1. Summary of the samples used in the current study

Table S2. List of synonyms substituted by accepted generic names in the current study

Figures

Fig. S1 Sampling locations and sequence counts for ITS1_OTU (red solid dots) and ITS2_OTU (blue solid triangle). The function sample.locations in R package RAM (1), a wrapper of functions in ggmap (2), was used to generate the figure, with Google Maps as the map source.

Fig. S2 Climatological data of sampling time points. The value of each variable was scaled to range 0 to 1. Tmax, maximum temperature; Tmin, minimum temperature; Tave, average temperature; CDD, Cooling Degree Days; HDD, Heating Degree Days; Ptot, total precipitation; Wdir, wind direction defined as the degree from north; Wmax, maximum wind speed. The wind data were missing for the sampling site at Saint−Clotilde (SC), Québec. The temperature-associated parameters assessed during the sampling weeks at each location were similar for 2010 and 2011, but the total precipitation and wind conditions were quite different. Ptot was higher at the beginning of the summer at all three locations, but during the summer, the east coast was wetter than the west coast. Wdir at Agassiz (west coast) was mainly from southwest to northeast (Wdir <= 90° from the north); while that of Harrington (east coast), was also mainly from southeast to northwest but occasionally from northwest to southeast (90 ^{o} <= Wdir <= 180 ^{o} from north). Wmax was much higher at Harrington than at Agassiz. Wind data were missing for

the sampling site at Saint-Clotilde (east inland city); therefore, samples collected from this location were removed when wind data were included in a model.

Fig. S3 The shift of diversity indices over the sampling weeks. Significant differences of the diversity indices between sampling weeks were annotated as: ***, p <= 0.001; **, p <= 0.01 and p > 0.001; *, p <= 0.05 and p > 0.01.

Fig. S4 The ratio of diversity indices of Ascomycota and Basidiomycota based on ITS1_OTU and ITS2_OTU. spec: specnumber (observed richness); chao: chao1 index (expected richness); sim_even: Gini-Simpson dominance evenness; shan_even: Shannon−Wiener evenness; sim_trudiv: Gini-Simpson true diversity; shan_trudiv: Shannon−Wiener true diversity.

Fig. S5 The observed relative abundance of selected genera recovered by each spore sampler through the sampling season: a) *Cladosporium* spp., b) *Drechslera* spp., c) *Entyloma* spp., d) *Fusarium* spp., e) *Microdochium* spp., f) *Ustilago* spp., g) *Exobasidium* spp., h) *Podosphaera* spp., i) *Ramularia* spp.

Fig. S6 Fungal taxa with number of OTUs recovered by ITS1 and ITS2 significantly (p <= 0.001) different at the A) class, B) order, C) family and D) genus levels.

References:

1. Chen W, Lévesque CA, Simpson J. 2017. RAM: R for amplicon-sequencing-based microbial-ecology, http://cran.r-project.org/package=RAM.
2. Kahle D, Wickham H. 2013. ggmap: Spatial Visualization with ggplot2. R Journal 5:144--161.

Supplementary Table 1. Summary of the samples used in the current study

	Sample_name	BioSample	BioProject	SRA_Study_ID	PlateMID	SFF	MID	Date	Sample	Year	Week	Province	City	Collector	Substrate	PCR_taq	PCR_ann	PCR_reps	PCR_cycle	Tmax	Tmin	Tave	HDD	CDD	Ptot	Wdir	Wmax	Latitude	Longitude	Elevation
BK185W01PE_T_1	GS3C5UA01.MID1	SAMN06167495	PRJNA358221	SRP095430	P3.1M1	GS3C5UA01	MID1	10-06-08	BK185W01PE	2010	1	PEI	Harrington	BK	Air	T	60	6	28	14.4625	8.575	11.5375	6.4625	0	110.1	19.07136958	154.3650702	46.34	-63.17	53
BK183W01BC_T_1	GS3C5UA01.MID10	SAMN06167496	PRJNA358221	SRP095430	P3.1M10	GS3C5UA01	MID10	10-06-08	BK183W01BC	2010	1	BC	Agassiz	BK	Air	T	59	12	26	18.5625	10.25	14.425	3.575	0	51.4	50	39	49.24	-121.76	19.3
BK183W01BC_T_2	GS3C5UA01.MID11	SAMN06167497	PRJNA358221	SRP095430	P3.1M11	GS3C5UA01	MID11	10-06-08	BK183W01BC	2010	1	BC	Agassiz	BK	Air	T	59	12	26	18.5625	10.25	14.425	3.575	0	51.4	50	39	49.24	-121.76	19.3
BK185W01PE_T_2	GS3C5UA01.MID2	SAMN06167498	PRJNA358221	SRP095430	P3.1M2	GS3C5UA01	MID2	10-06-08	BK185W01PE	2010	1	PEI	Harrington	BK	Air	T	60	6	28	14.4625	8.575	11.5375	6.4625	0	110.1	19.07136958	154.3650702	46.34	-63.17	53
BK182W01SC_T_1	GS3C5UA01.MID4	SAMN06167499	PRJNA358221	SRP095430	P3.1M4	GS3C5UA01	MID4	10-06-08	BK182W01SC	2010	1	QC	SainteClotilde	BK	Air	T	60	6	22	21.75	12.275	17.05	1.6375	0.6875	85.9	NA	NA	45.08	-73.38	49.3
BK182W01SC_T_2	GS3C5UA01.MID5	SAMN06167500	PRJNA358221	SRP095430	P3.1M5	GS3C5UA01	MID5	10-06-08	BK182W01SC	2010	1	QC	SainteClotilde	BK	Air	T	60	6	22	21.75	12.275	17.05	1.6375	0.6875	85.9	NA	NA	45.08	-73.38	49.3
BK185W01PE_P_1	GS3C5UA02.MID13	SAMN06167501	PRJNA358221	SRP095430	P3.2M13	GS3C5UA02	MID13	10-06-08	BK185W01PE	2010	1	PEI	Harrington	BK	Air	P	56	8	32	14.4625	8.575	11.5375	6.4625	0	110.1	19.07136958	154.3650702	46.34	-63.17	53
BK185W01PE_P_2	GS3C5UA02.MID14	SAMN06167502	PRJNA358221	SRP095430	P3.2M14	GS3C5UA02	MID14	10-06-08	BK185W01PE	2010	1	PEI	Harrington	BK	Air	P	56	8	32	14.4625	8.575	11.5375	6.4625	0	110.1	19.07136958	154.3650702	46.34	-63.17	53
BK182W01SC_P_1	GS3C5UA02.MID16	SAMN06167503	PRJNA358221	SRP095430	P3.2M16	GS3C5UA02	MID16	10-06-08	BK182W01SC	2010	1	QC	SainteClotilde	BK	Air	P	56	8	25	21.75	12.275	17.05	1.6375	0.6875	85.9	NA	NA	45.08	-73.38	49.3
BK182W01SC_P_2	GS3C5UA02.MID17	SAMN06167504	PRJNA358221	SRP095430	P3.2M17	GS3C5UA02	MID17	10-06-08	BK182W01SC	2010	1	QC	SainteClotilde	BK	Air	P	56	8	25	21.75	12.275	17.05	1.6375	0.6875	85.9	NA	NA	45.08	-73.38	49.3
BK183W01BC_P_1	GS3C5UA02.MID22	SAMN06167505	PRJNA358221	SRP095430	P3.2M22	GS3C5UA02	MID22	10-06-08	BK183W01BC	2010	1	BC	Agassiz	BK	Air	P	56	8	25	18.5625	10.25	14.425	3.575	0	51.4	50	39	49.24	-121.76	19.3
BK183W01BC_P_2	GS3C5UA02.MID23	SAMN06167506	PRJNA358221	SRP095430	P3.2M23	GS3C5UA02	MID23	10-06-08	BK183W01BC	2010	1	BC	Agassiz	BK	Air	P	56	8	25	18.5625	10.25	14.425	3.575	0	51.4	50	39	49.24	-121.76	19.3
YE028W01PE_P	GX8ARDE01.MID1	SAMN06167507	PRJNA358221	SRP095430	P4.1M1	GX8ARDE01	MID1	10-06-08	YE028W01PE	2010	1	PEI	Harrington	YE	Rain	P	56	8	25	14.4625	8.575	11.5375	6.4625	0	110.1	19.07136958	154.3650702	46.34	-63.17	53
YE026W01BC_P	GX8ARDE01.MID10	SAMN06167508	PRJNA358221	SRP095430	P4.1M10	GX8ARDE01	MID10	10-06-08	YE026W01BC	2010	1	BC	Agassiz	YE	Rain	P	56	8	35	18.5625	10.25	14.425	3.575	0	51.4	50	39	49.24	-121.76	19.3
JB888W01BC_P	GX8ARDE01.MID11	SAMN06167509	PRJNA358221	SRP095430	P4.1M11	GX8ARDE01	MID11	10-06-08	JB888W01BC	2010	1	BC	Agassiz	JB	Rain	P	56	8	35	18.5625	10.25	14.425	3.575	0	51.4	50	39	49.24	-121.76	19.3
JB895W01PE_P	GX8ARDE01.MID2	SAMN06167510	PRJNA358221	SRP095430	P4.1M2	GX8ARDE01	MID2	10-06-08	JB895W01PE	2010	1	PEI	Harrington	JB	Rain	P	56	8	30	14.4625	8.575	11.5375	6.4625	0	110.1	19.07136958	154.3650702	46.34	-63.17	53
YE025W01SC_P	GX8ARDE01.MID3	SAMN06167511	PRJNA358221	SRP095430	P4.1M3	GX8ARDE01	MID3	10-06-08	YE025W01SC	2010	1	QC	SainteClotilde	YE	Rain	P	56	8	40	21.75	12.275	17.05	1.6375	0.6875	85.9	NA	NA	45.08	-73.38	49.3
JB886W01SC_P	GX8ARDE01.MID4	SAMN06167512	PRJNA358221	SRP095430	P4.1M4	GX8ARDE01	MID4	10-06-08	JB886W01SC	2010	1	QC	SainteClotilde	JB	Rain	P	56	8	30	21.75	12.275	17.05	1.6375	0.6875	85.9	NA	NA	45.08	-73.38	49.3
JB958W05BC_P	G2W0QXL01.MID3	SAMN06167466	PRJNA358221	SRP095430	P6.1M3	G2W0QXL01	MID3	10-07-06	JB958W05BC	2010	5	BC	Agassiz	JB	Rain	P	56	8	28	19.55	11.3	15.4625	2.625	0.0875	2	0	0	49.24	-121.76	19.3
BK208W05BC_P	G2W0QXL01.MID4	SAMN06167467	PRJNA358221	SRP095430	P6.1M4	G2W0QXL01	MID4	10-07-06	BK208W05BC	2010	5	BC	Agassiz	BK	Air	P	56	8	28	19.55	11.3	15.4625	2.625	0.0875	2	0	0	49.24	-121.76	19.3
YE044W05PE_P	GX8ARDE02.MID14	SAMN06167513	PRJNA358221	SRP095430	P4.2M14	GX8ARDE02	MID14	10-07-06	YE044W05PE	2010	5	PEI	Harrington	YE	Rain	P	56	8	25	21.575	13.575	17.6125	1.275	0.8875	3.7	15.7774004	148.3098849	46.34	-63.17	53
JB959W05PE_P	GX8ARDE02.MID15	SAMN06167514	PRJNA358221	SRP095430	P4.2M15	GX8ARDE02	MID15	10-07-06	JB959W05PE	2010	5	PEI	Harrington	JB	Rain	P	56	8	25	21.575	13.575	17.6125	1.275	0.8875	3.7	15.7774004	148.3098849	46.34	-63.17	53
BK212W05PE_P	GX8ARDE02.MID16	SAMN06167515	PRJNA358221	SRP095430	P4.2M16	GX8ARDE02	MID16	10-07-06	BK212W05PE	2010	5	PEI	Harrington	BK	Air	P	56	8	35	21.575	13.575	17.6125	1.275	0.8875	3.7	15.7774004	148.3098849	46.34	-63.17	53
YE045W05SC_P	GX8ARDE02.MID17	SAMN06167516	PRJNA358221	SRP095430	P4.2M17	GX8ARDE02	MID17	10-07-06	YE045W05SC	2010	5	QC	SainteClotilde	YE	Rain	P	56	8	40	27.375	15.075	21.2375	0.55	3.7875	6.7	NA	NA	45.08	-73.38	49.3
JB960W05SC_P	GX8ARDE02.MID18	SAMN06167517	PRJNA358221	SRP095430	P4.2M18	GX8ARDE02	MID18	10-07-06	JB960W05SC	2010	5	QC	SainteClotilde	JB	Rain	P	56	8	35	26.625	14.3375	20.5	0.55	3.05	6.7	NA	NA	45.08	-73.38	49.3
BK213W05SC_P	GX8ARDE02.MID19	SAMN06167518	PRJNA358221	SRP095430	P4.2M19	GX8ARDE02	MID19	10-07-06	BK213W05SC	2010	5	QC	SainteClotilde	BK	Air	P	56	8	40	26.625	14.3375	20.5	0.55	3.05	6.7	NA	NA	45.08	-73.38	49.3
JB1026W09PE_P	G2W0QXL01.MID10	SAMN06167463	PRJNA358221	SRP095430	P6.1M10	G2W0QXL01	MID10	10-08-03	JB1026W09PE	2010	9	PEI	Harrington	JB	Rain	P	56	8	40	22.3875	13	17.7125	0.9125	0.625	2.8	51.82066186	118.416112	46.34	-63.17	53
BK241W09PE_P	G2W0QXL01.MID11	SAMN06167464	PRJNA358221	SRP095430	P6.1M11	G2W0QXL01	MID11	10-08-03	BK241W09PE	2010	9	PEI	Harrington	BK	Air	P	56	8	28	22.3875	13	17.7125	0.9125	0.625	2.8	51.82066186	118.416112	46.34	-63.17	53
JB1015W09SC_P	G2W0QXL01.MID12	SAMN06167465	PRJNA358221	SRP095430	P6.1M12	G2W0QXL01	MID12	10-08-03	JB1015W09SC	2010	9	QC	SainteClotilde	JB	Rain	P	56	16	40	26.0875	13.7125	19.9375	0.425	2.3625	44.8	NA	NA	45.08	-73.38	49.3
YE061W09PE_P	G2W0QXL01.MID9	SAMN06167468	PRJNA358221	SRP095430	P6.1M9	G2W0QXL01	MID9	10-08-03	YE061W09PE	2010	9	PEI	Harrington	YE	Rain	P	56	8	28	22.3875	13	17.7125	0.9125	0.625	2.8	51.82066186	118.416112	46.34	-63.17	53
BK234W09SC_P	G2W0QXL02.MID13	SAMN06167469	PRJNA358221	SRP095430	P6.2M13	G2W0QXL02	MID13	10-08-03	BK234W09SC	2010	9	QC	SainteClotilde	BK	Air	P	56	8	40	26.0875	13.7125	19.9375	0.425	2.3625	44.8	NA	NA	45.08	-73.38	49.3
JB1010W09BC_P	G2W0QXL02.MID22	SAMN06167470	PRJNA358221	SRP095430	P6.2M22	G2W0QXL02	MID22	10-08-03	JB1010W09BC	2010	9	BC	Agassiz	JB	Rain	P	56	8	40	25.625	12.8125	19.2375	0.1375	1.375	7.4	40	41	49.24	-121.76	19.3
BK233W09BC_P	G2W0QXL02.MID23	SAMN06167471	PRJNA358221	SRP095430	P6.2M23	G2W0QXL02	MID23	10-08-03	BK233W09BC	2010	9	BC	Agassiz	BK	Air	P	56	8	28	25.625	12.8125	19.2375	0.1375	1.375	7.4	40	41	49.24	-121.76	19.3
YE074W13PE_P	G4L2DRQ01.MID2	SAMN06167472	PRJNA358221	SRP095430	P8.1M2	G4L2DRQ01	MID2	10-08-31	YE074W13PE	2010	13	PEI	Harrington	YE	Rain	P	56	8	28	23.5	14.4625	19	0.275	1.275	41.7	40.74438549	105.3049116	46.34	-63.17	53
JB1079W13PE_P	G4L2DRQ01.MID3	SAMN06167473	PRJNA358221	SRP095430	P8.1M3	G4L2DRQ01	MID3	10-08-31	JB1079W13PE	2010	13	PEI	Harrington	JB	Rain	P	56	8	28	23.5	14.4625	19	0.275	1.275	41.7	40.74438549	105.3049116	46.34	-63.17	53
BK265W13PE_P	G4L2DRQ01.MID4	SAMN06167474	PRJNA358221	SRP095430	P8.1M4	G4L2DRQ01	MID4	10-08-31	BK265W13PE	2010	13	PEI	Harrington	BK	Air	P	56	8	28	23.5	14.4625	19	0.275	1.275	41.7	40.74438549	105.3049116	46.34	-63.17	53
YE076W13SC_P	G4L2DRQ01.MID5	SAMN06167475	PRJNA358221	SRP095430	P8.1M5	G4L2DRQ01	MID5	10-08-31	YE076W13SC	2010	13	QC	SainteClotilde	YE	Rain	P	56	8	28	27.3625	13.875	20.6375	0.125	2.7625	0.8	NA	NA	45.08	-73.38	49.3
JB1086W13SC_P	G4L2DRQ01.MID6	SAMN06167476	PRJNA358221	SRP095430	P8.1M6	G4L2DRQ01	MID6	10-08-31	JB1086W13SC	2010	13	QC	SainteClotilde	JB	Rain	P	56	8	28	27.3625	13.875	20.6375	0.125	2.7625	0.8	NA	NA	45.08	-73.38	49.3
BK267W13SC_P	G4L2DRQ01.MID7	SAMN06167477	PRJNA358221	SRP095430	P8.1M7	G4L2DRQ01	MID7	10-08-31	BK267W13SC	2010	13	QC	SainteClotilde	BK	Air	P	56	10	52	27.3625	13.875	20.6375	0.125	2.7625	0.8	NA	NA	45.08	-73.38	49.3
YE073W13BC_P	G4L2DRQ02.MID16	SAMN06167478	PRJNA358221	SRP095430	P8.2M16	G4L2DRQ02	MID16	10-08-31	YE073W13BC	2010	13	BC	Agassiz	YE	Rain	P	56	8	28	22.3375	11.025	16.7125	2.05	0.7625	28.8	50.19905119	99.96753048	49.24	-121.76	19.3
JB1074W13BC_P	G4L2DRQ02.MID17	SAMN06167479	PRJNA358221	SRP095430	P8.2M17	G4L2DRQ02	MID17	10-08-31	JB1074W13BC	2010	13	BC	Agassiz	JB	Rain	P	56	8	28	22.3375	11.025	16.7125	2.05	0.7625	28.8	50.19905119	99.96753048	49.24	-121.76	19.3
BK260W13BC_P	G4L2DRQ02.MID18	SAMN06167480	PRJNA358221	SRP095430	P8.2M18	G4L2DRQ02	MID18	10-08-31	BK260W13BC	2010	13	BC	Agassiz	BK	Air	P	56	8	28	22.3375	11.025	16.7125	2.05	0.7625	28.8	50.19905119	99.96753048	49.24	-121.76	19.3
YE094W17PE_P	G4L2DRQ02.MID23	SAMN06167481	PRJNA358221	SRP095430	P8.2M23	G4L2DRQ02	MID23	10-09-28	YE094W17PE	2010	17	PEI	Harrington	YE	Rain	P	56	8	35	16.275	7.775	12.0625	5.9375	0	12.2	61.53195415	245.8359073	46.34	-63.17	53
JB1135W17PE_P	G4L2DRQ02.MID24	SAMN06167482	PRJNA358221	SRP095430	P8.2M24	G4L2DRQ02	MID24	10-09-28	JB1135W17PE	2010	17	PEI	Harrington	JB	Rain	P	56	8	35	16.275	7.775	12.0625	5.9375	0	12.2	61.53195415	245.8359073	46.34	-63.17	53
BK291W17PE_P	G5SJEKS01.MID1	SAMN06167483	PRJNA358221	SRP095430	P10.1M1	G5SJEKS01	MID1	10-09-28	BK291W17PE	2010	17	PEI	Harrington	BK	Air	P	56	8	28	16.275	7.775	12.0625	5.9375	0	12.2	61.53195415	245.8359073	46.34	-63.17	53
YE090W17SC_P	G5SJEKS01.MID2	SAMN06167484	PRJNA358221	SRP095430	P10.1M2	G5SJEKS01	MID2	10-09-28	YE090W17SC	2010	17	QC	SainteClotilde	YE	Rain	P	56	8	28	19.88571429	10.15714286	14.95	3.533333333	0.483333333	41.9	NA	NA	45.08	-73.38	49.3
JB1126W17SC_P	G5SJEKS01.MID3	SAMN06167485	PRJNA358221	SRP095430	P10.1M3	G5SJEKS01	MID3	10-09-28	JB1126W17SC	2010	17	QC	SainteClotilde	JB	Rain	P	56	8	28	19.88571429	10.15714286	14.95	3.533333333	0.483333333	41.9	NA	NA	45.08	-73.38	49.3
BK284W17SC_P	G5SJEKS01.MID4	SAMN06167486	PRJNA358221	SRP095430	P10.1M4	G5SJEKS01	MID4	10-09-28	BK284W17SC	2010	17	QC	SainteClotilde	BK	Air	P	56	8	28	19.88571429	10.15714286	14.95	3.533333333	0.483333333	41.9	NA	NA	45.08	-73.38	49.3
YE092W17BC_P	G5SJEKS02.MID14	SAMN06167487	PRJNA358221	SRP095430	P10.2M14	G5SJEKS02	MID14	10-09-28	YE092W17BC	2010	17	BC	Agassiz	YE	Rain	P	56	8	28	19.25	11.25	15.25	2.75	0	63.2	55.9108086	116.1403335	49.24	-121.76	19.3
JB1131W17BC_P	G5SJEKS02.MID15	SAMN06167488	PRJNA358221	SRP095430	P10.2M15	G5SJEKS02	MID15	10-09-28	JB1131W17BC	2010	17	BC	Agassiz	JB	Rain	P	56	11	40	19.25	11.25	15.25	2.75	0	63.2	55.9108086	116.1403335	49.24	-121.76	19.3
BK286W17BC_P	G5SJEKS02.MID16	SAMN06167489	PRJNA358221	SRP095430	P10.2M16	G5SJEKS02	MID16	10-09-28	BK286W17BC	2010	17	BC	Agassiz	BK	Air	P	56	8	28	19.25	11.25	15.25	2.75	0	63.2	55.9108086	116.1403335	49.24	-121.76	19.3
YE101W01PE_P	G9CKJAV02.MID14	SAMN06167490	PRJNA358221	SRP095430	P18.2M14	G9CKJAV02	MID14	11-06-07	YE101W01PE	2011	1	PEI	Harrington	YE	Rain	P	60	8	35	14.225	7	10.65	7.35	0	6.9	42.45386553	125.0411909	46.34	-63.17	53
JB1151W01PE_P	G9CKJAV02.MID15	SAMN06167491	PRJNA358221	SRP095430	P18.2M15	G9CKJAV02	MID15	11-06-07	JB1151W01PE	2011	1	PEI	Harrington	JB	Rain	P	60	8	35	14.225	7	10.65	7.35	0	6.9	42.45386553	125.0411909	46.34	-63.17	53
BK300W01PE_P	G9CKJAV02.MID16	SAMN06167492	PRJNA358221	SRP095430	P18.2M16	G9CKJAV02	MID16	11-06-07	BK300W01PE	2011	1	PEI	Harrington	BK	Air	P	60	8	35	14.225	7	10.65	7.35	0	6.9	42.45386553	125.0411909	46.34	-63.17	53
JB1162W01SC_P	G9CKJAV02.MID17	SAMN06167493	PRJNA358221	SRP095430	P18.2M17	G9CKJAV02	MID17	11-06-07	JB1162W01SC	2011	1	QC	SainteClotilde	JB	Rain	P	60	8	35	24.94285714	12.9	20.7	1.025	3.725	0.5	NA	NA	45.08	-73.38	49.3
BK303W01SC_T	G9CKJAV02.MID18	SAMN06167494	PRJNA358221	SRP095430	P18.2M18	G9CKJAV02	MID18	11-06-07	BK303W01SC	2011	1	QC	SainteClotilde	BK	Air	T	60	16	30	24.94285714	12.9	20.7	1.025	3.725	0.5	NA	NA	45.08	-73.38	49.3
YE100W01BC_P	HAT26ZH01.MID2	SAMN06167522	PRJNA358221	SRP095430	P20.1M2	HAT26ZH01	MID2	11-06-07	YE100W01BC	2011	1	BC	Agassiz	YE	Rain	P	60	8	35	20.325	11.125	15.7375	2.4625	0.2	29	0	0	49.24	-121.76	19.3
JB1150W01BC_P	HAT26ZH01.MID3	SAMN06167523	PRJNA358221	SRP095430	P20.1M3	HAT26ZH01	MID3	11-06-07	JB1150W01BC	2011	1	BC	Agassiz	JB	Rain	P	60	8	35	20.325	11.125	15.7375	2.4625	0.2	29	0	0	49.24	-121.76	19.3
BK307W01BC_P	HAT26ZH01.MID4	SAMN06167524	PRJNA358221	SRP095430	P20.1M4	HAT26ZH01	MID4	11-06-07	BK307W01BC	2011	1	BC	Agassiz	BK	Air	P	60	8	35	20.325	11.125	15.7375	2.4625	0.2	29	0	0	49.24	-121.76	19.3
BK332W05PE_P	HAT26ZH01.MID10	SAMN06167519	PRJNA358221	SRP095430	P20.1M10	HAT26ZH01	MID10	11-07-05	BK332W05PE	2011	5	PEI	Harrington	BK	Air	P	60	8	35	23.1625	13.675	18.4625	0.4	0.8625	7.7	40.49251442	65.23320999	46.34	-63.17	53
YE118W05SC_P	HAT26ZH01.MID11	SAMN06167520	PRJNA358221	SRP095430	P20.1M11	HAT26ZH01	MID11	11-07-05	YE118W05SC	2011	5	QC	SainteClotilde	YE	Rain	P	60	8	35	26.6375	16.07142857	21.71428571	0.071428571	3.785714286	8.1	NA	NA	45.08	-73.38	49.3
JB1227W05SC_P	HAT26ZH01.MID12	SAMN06167521	PRJNA358221	SRP095430	P20.1M12	HAT26ZH01	MID12	11-07-05	JB1227W05SC	2011	5	QC	SainteClotilde	JB	Rain	P	60	8	35	26.6375	16.07142857	21.71428571	0.071428571	3.785714286	8.1	NA	NA	45.08	-73.38	49.3
YE116W05PE_T	HAT26ZH01.MID8	SAMN06167525	PRJNA358221	SRP095430	P20.1M8	HAT26ZH01	MID8	11-07-05	YE116W05PE	2011	5	PEI	Harrington	YE	Rain	T	60	8	30	23.1625	13.675	18.4625	0.4	0.8625	7.7	40.49251442	65.23320999	46.34	-63.17	53
JB1221W05PE_T	HAT26ZH01.MID9	SAMN06167526	PRJNA358221	SRP095430	P20.1M9	HAT26ZH01	MID9	11-07-05	JB1221W05PE	2011	5	PEI	Harrington	JB	Rain	T	60	8	30	23.1625	13.675	18.4625	0.4	0.8625	7.7	40.49251442	65.23320999	46.34	-63.17	53
BK334W05SC_P	HAT26ZH02.MID13	SAMN06167527	PRJNA358221	SRP095430	P20.2M13	HAT26ZH02	MID13	11-07-05	BK334W05SC	2011	5	QC	SainteClotilde	BK	Air	P	60	8	35	26.6375	16.07142857	21.71428571	0.071428571	3.785714286	8.1	NA	NA	45.08	-73.38	49.3
YE115W05BC_P	HAT26ZH02.MID23	SAMN06167528	PRJNA358221	SRP095430	P20.2M23	HAT26ZH02	MID23	11-07-05	YE115W05BC	2011	5	BC	Agassiz	YE	Rain	P	60	8	35	21.9	11.575	16.775	1.4375	0.2125	35.6	30	74	49.24	-121.76	19.3
JB1220W05BC_P	HAT26ZH02.MID24	SAMN06167529	PRJNA358221	SRP095430	P20.2M24	HAT26ZH02	MID24	11-07-05	JB1220W05BC	2011	5	BC	Agassiz	JB	Rain	P	60	8	35	21.9	11.575	16.775	1.4375	0.2125	35.6	30	74	49.24	-121.76	19.3
BK328W05BC_P	HB8DEAV01.MID2	SAMN06167533	PRJNA358221	SRP095430	P22.1M2	HB8DEAV01	MID2	11-07-05	BK328W05BC	2011	5	BC	Agassiz	BK	Air	P	60	8	35	21.9	11.575	16.775	1.4375	0.2125	35.6	30	74	49.24	-121.76	19.3
YE135W09SC_T	HB8DEAV01.MID10	SAMN06167530	PRJNA358221	SRP095430	P22.1M10	HB8DEAV01	MID10	11-08-02	YE135W09SC	2011	9	QC	SainteClotilde	YE	Rain	T	60	8	30	27.24285714	15.1625	21.22857143	0	3.228571429	26.5	NA	NA	45.08	-73.38	49.3
JB1300W09SC_P	HB8DEAV01.MID11	SAMN06167531	PRJNA358221	SRP095430	P22.1M11	HB8DEAV01	MID11	11-08-02	JB1300W09SC	2011	9	QC	SainteClotilde	JB	Rain	P	60	8	35	27.24285714	15.1625	21.22857143	0	3.228571429	26.5	NA	NA	45.08	-73.38	49.3
BK363W09SC_T	HB8DEAV01.MID12	SAMN06167532	PRJNA358221	SRP095430	P22.1M12	HB8DEAV01	MID12	11-08-02	BK363W09SC	2011	9	QC	SainteClotilde	BK	Air	T	60	8	30	27.24285714	15.1625	21.22857143	0	3.228571429	26.5	NA	NA	45.08	-73.38	49.3
YE133W09PE_P	HB8DEAV01.MID7	SAMN06167534	PRJNA358221	SRP095430	P22.1M7	HB8DEAV01	MID7	11-08-02	YE133W09PE	2011	9	PEI	Harrington	YE	Rain	P	60	8	35	20.95	13.825	17.4125	0.775	0.1875	85.6	7.951697607	86.92473436	46.34	-63.17	53
JB1296W09PE_P	HB8DEAV01.MID8	SAMN06167535	PRJNA358221	SRP095430	P22.1M8	HB8DEAV01	MID8	11-08-02	JB1296W09PE	2011	9	PEI	Harrington	JB	Rain	P	60	8	35	20.95	13.825	17.4125	0.775	0.1875	85.6	7.951697607	86.92473436	46.34	-63.17	53
BK361W09PE_T	HB8DEAV01.MID9	SAMN06167536	PRJNA358221	SRP095430	P22.1M9	HB8DEAV01	MID9	11-08-02	BK361W09PE	2011	9	PEI	Harrington	BK	Air	T	60	8	30	20.95	13.825	17.4125	0.775	0.1875	85.6	7.951697607	86.92473436	46.34	-63.17	53
YE130W09BC_P	HB8DEAV02.MID22	SAMN06167537	PRJNA358221	SRP095430	P22.2M22	HB8DEAV02	MID22	11-08-02	YE130W09BC	2011	9	BC	Agassiz	YE	Rain	P	60	8	35	23.6	12.775	18.225	0.5375	0.7625	6	40	32	49.24	-121.76	19.3
JB1288W09BC_P	HB8DEAV02.MID23	SAMN06167538	PRJNA358221	SRP095430	P22.2M23	HB8DEAV02	MID23	11-08-02	JB1288W09BC	2011	9	BC	Agassiz	JB	Rain	P	60	8	35	23.6	12.775	18.225	0.5375	0.7625	6	40	32	49.24	-121.76	19.3
BK355W09BC_P	HB8DEAV02.MID24	SAMN06167539	PRJNA358221	SRP095430	P22.2M24	HB8DEAV02	MID24	11-08-02	BK355W09BC	2011	9	BC	Agassiz	BK	Air	P	60	8	35	23.6	12.775	18.225	0.5375	0.7625	6	40	32	49.24	-121.76	19.3
BK387W13SC_P	HD2UIFH01.MID10	SAMN06167540	PRJNA358221	SRP095430	P24.3M10	HD2UIFH01	MID10	11-08-30	BK387W13SC	2011	13	QC	SainteClotilde	BK	Air	P	60	8	30	23.71111111	13.46	18.53333333	0.511111111	1.044444444	141.7	NA	NA	45.08	-73.38	49.3
YE148W13PE_P	HD2UIFH01.MID5	SAMN06167541	PRJNA358221	SRP095430	P24.3M5	HD2UIFH01	MID5	11-08-30	YE148W13PE	2011	13	PEI	Harrington	YE	Rain	P	60	8	30	24.0375	14.1625	19.125	0.25	1.375	25.2	136.8017111	80.42758423	46.34	-63.17	53
JB1352W13PE_P	HD2UIFH01.MID6	SAMN06167542	PRJNA358221	SRP095430	P24.3M6	HD2UIFH01	MID6	11-08-30	JB1352W13PE	2011	13	PEI	Harrington	JB	Rain	P	60	8	30	24.0375	14.1625	19.125	0.25	1.375	25.2	136.8017111	80.42758423	46.34	-63.17	53
BK383W13PE_P	HD2UIFH01.MID7	SAMN06167543	PRJNA358221	SRP095430	P24.3M7	HD2UIFH01	MID7	11-08-30	BK383W13PE	2011	13	PEI	Harrington	BK	Air	P	60	8	30	24.0375	14.1625	19.125	0.25	1.375	25.2	136.8017111	80.42758423	46.34	-63.17	53
JB1366W13SC_P	HD2UIFH01.MID9	SAMN06167544	PRJNA358221	SRP095430	P24.3M9	HD2UIFH01	MID9	11-08-30	JB1366W13SC	2011	13	QC	SainteClotilde	JB	Rain	P	60	8	30	23.16666667	13.28571429	18.11666667	0.766666667	0.883333333	141.5	NA	NA	45.08	-73.38	49.3
JB1369W13BC_P	HD2UIFH02.MID19	SAMN06167545	PRJNA358221	SRP095430	P24.4M19	HD2UIFH02	MID19	11-08-30	JB1369W13BC	2011	13	BC	Agassiz	JB	Rain	P	60	8	30	25.7625	13.3875	19.5875	0.25	1.8375	2.2	50	32	49.24	-121.76	19.3
BK391W13BC_P	HD2UIFH02.MID20	SAMN06167546	PRJNA358221	SRP095430	P24.4M20	HD2UIFH02	MID20	11-08-30	BK391W13BC	2011	13	BC	Agassiz	BK	Air	P	60	8	30	25.7625	13.3875	19.5875	0.25	1.8375	2.2	50	32	49.24	-121.76	19.3
YE164W17BC_P	HI17QTS02.MID15	SAMN06167547	PRJNA358221	SRP095430	P26.2M15	HI17QTS02	MID15	11-09-27	YE164W17BC	2011	17	BC	Agassiz	YE	Rain	P	60	8	30	20.675	11.725	16.225	2.2375	0.4625	130	38.96431104	170.2986206	49.24	-121.76	19.3
JB1418W17BC_P	HI17QTS02.MID16	SAMN06167548	PRJNA358221	SRP095430	P26.2M16	HI17QTS02	MID16	11-09-27	JB1418W17BC	2011	17	BC	Agassiz	JB	Rain	P	60	8	30	20.675	11.725	16.225	2.2375	0.4625	130	38.96431104	170.2986206	49.24	-121.76	19.3
BK416W17BC_P	HI17QTS02.MID17	SAMN06167549	PRJNA358221	SRP095430	P26.2M17	HI17QTS02	MID17	11-09-27	BK416W17BC	2011	17	BC	Agassiz	BK	Air	P	60	8	30	20.675	11.725	16.225	2.2375	0.4625	130	38.96431104	170.2986206	49.24	-121.76	19.3
JB1412W17PE_P_1	HOVO39H01.MID3	SAMN06167550	PRJNA358221	SRP095430	P30.3M3	HOVO39H01	MID3	11-09-27	JB1412W17PE	2011	17	PEI	Harrington	JB	Rain	P	60	8	38	20.125	11.5375	15.8625	2.5375	0.4	0.5	37.87143652	144.0682887	46.34	-63.17	53
BK411W17PE_P_1	HOVO39H01.MID4	SAMN06167551	PRJNA358221	SRP095430	P30.3M4	HOVO39H01	MID4	11-09-27	BK411W17PE	2011	17	PEI	Harrington	BK	Air	P	60	8	30	20.125	11.5375	15.8625	2.5375	0.4	0.5	37.87143652	144.0682887	46.34	-63.17	53
YE166W17SC_P_1	HOVO39H01.MID5	SAMN06167552	PRJNA358221	SRP095430	P30.3M5	HOVO39H01	MID5	11-09-27	YE166W17SC	2011	17	QC	SainteClotilde	YE	Rain	P	60	8	30	23.325	12.1375	17.775	0.9125	0.6875	9.1	NA	NA	45.08	-73.38	49.3
JB1424W17SC_P_1	HOVO39H01.MID6	SAMN06167553	PRJNA358221	SRP095430	P30.3M6	HOVO39H01	MID6	11-09-27	JB1424W17SC	2011	17	QC	SainteClotilde	JB	Rain	P	60	8	30	23.325	12.1375	17.775	0.9125	0.6875	9.1	NA	NA	45.08	-73.38	49.3
BK418W17SC_P_1	HOVO39H01.MID7	SAMN06167554	PRJNA358221	SRP095430	P30.3M7	HOVO39H01	MID7	11-09-27	BK418W17SC	2011	17	QC	SainteClotilde	BK	Air	P	60	8	30	23.325	12.1375	17.775	0.9125	0.6875	9.1	NA	NA	45.08	-73.38	49.3
JB1412W17PE_P_2	HR0LN3Q04.MID3	SAMN06167555	PRJNA358221	SRP095430	P30.6M3	HR0LN3Q04	MID3	11-09-27	JB1412W17PE	2011	17	PEI	Harrington	JB	Rain	P	60	8	38	20.125	11.5375	15.8625	2.5375	0.4	0.5	37.87143652	144.0682887	46.34	-63.17	53
BK411W17PE_P_2	HR0LN3Q04.MID4	SAMN06167556	PRJNA358221	SRP095430	P30.6M4	HR0LN3Q04	MID4	11-09-27	BK411W17PE	2011	17	PEI	Harrington	BK	Air	P	60	8	30	20.125	11.5375	15.8625	2.5375	0.4	0.5	37.87143652	144.0682887	46.34	-63.17	53
YE166W17SC_P_2	HR0LN3Q04.MID5	SAMN06167557	PRJNA358221	SRP095430	P30.6M5	HR0LN3Q04	MID5	11-09-27	YE166W17SC	2011	17	QC	SainteClotilde	YE	Rain	P	60	8	30	23.325	12.1375	17.775	0.9125	0.6875	9.1	NA	NA	45.08	-73.38	49.3
JB1424W17SC_P_2	HR0LN3Q04.MID6	SAMN06167558	PRJNA358221	SRP095430	P30.6M6	HR0LN3Q04	MID6	11-09-27	JB1424W17SC	2011	17	QC	SainteClotilde	JB	Rain	P	60	8	30	23.325	12.1375	17.775	0.9125	0.6875	9.1	NA	NA	45.08	-73.38	49.3
BK418W17SC_P_2	HR0LN3Q04.MID7	SAMN06167559	PRJNA358221	SRP095430	P30.6M7	HR0LN3Q04	MID7	11-09-27	BK418W17SC	2011	17	QC	SainteClotilde	BK	Air	P	60	8	30	23.325	12.1375	17.775	0.9125	0.6875	9.1	NA	NA	45.08	-73.38	49.3

Table S2. List of synonyms substituted by accepted generic names in the current study

Accepted generic name	Synonyms being substituted
Alternaria	Lewia
Ascocoryne	Coryne
Aspergillus	Eurotium, Emericella
Asterophora	Ugola
Bipolaris	Cochliobolus
Ceratobasidium	Ceratorhiza
Cladosporium	Davidiella
Claviceps	Sphacelia
Coprinellus	Ozonium
Coprinopsis	Hormographiella
Cristulariella	Nervostroma
Dendrocollybia	Tilachlidiopsis
Drechslera	Pyrenophora, Setosphaeria
Epichloe	Neotyphodium
Exobasidium	Tilletiopsis
Exserohilum	Setosphaeria
Fusarium	Gibberella
Ganoderma	Thermophymatospora
Grovesinia	Hinomyces
Hohenbuehelia	Nematoctonus
Microdochium	Monographella
Morchella	Costantinella
Nectria	Tubercularia
Onygena	Sporendonema
Penicillium	Eupenicillium
Pleurotus	Antromycopsis
Ramularia	Mycosphaerella
Termitomyces	Termitosphaera
Tolypocladium	Elaphocordyceps
Trichoderma	Hypocrea
Tulasnella	Epulorhiza
Ustilago	Pseudozyma
Valdensia	Valdensinia
Wolfiporia	Gemmularia

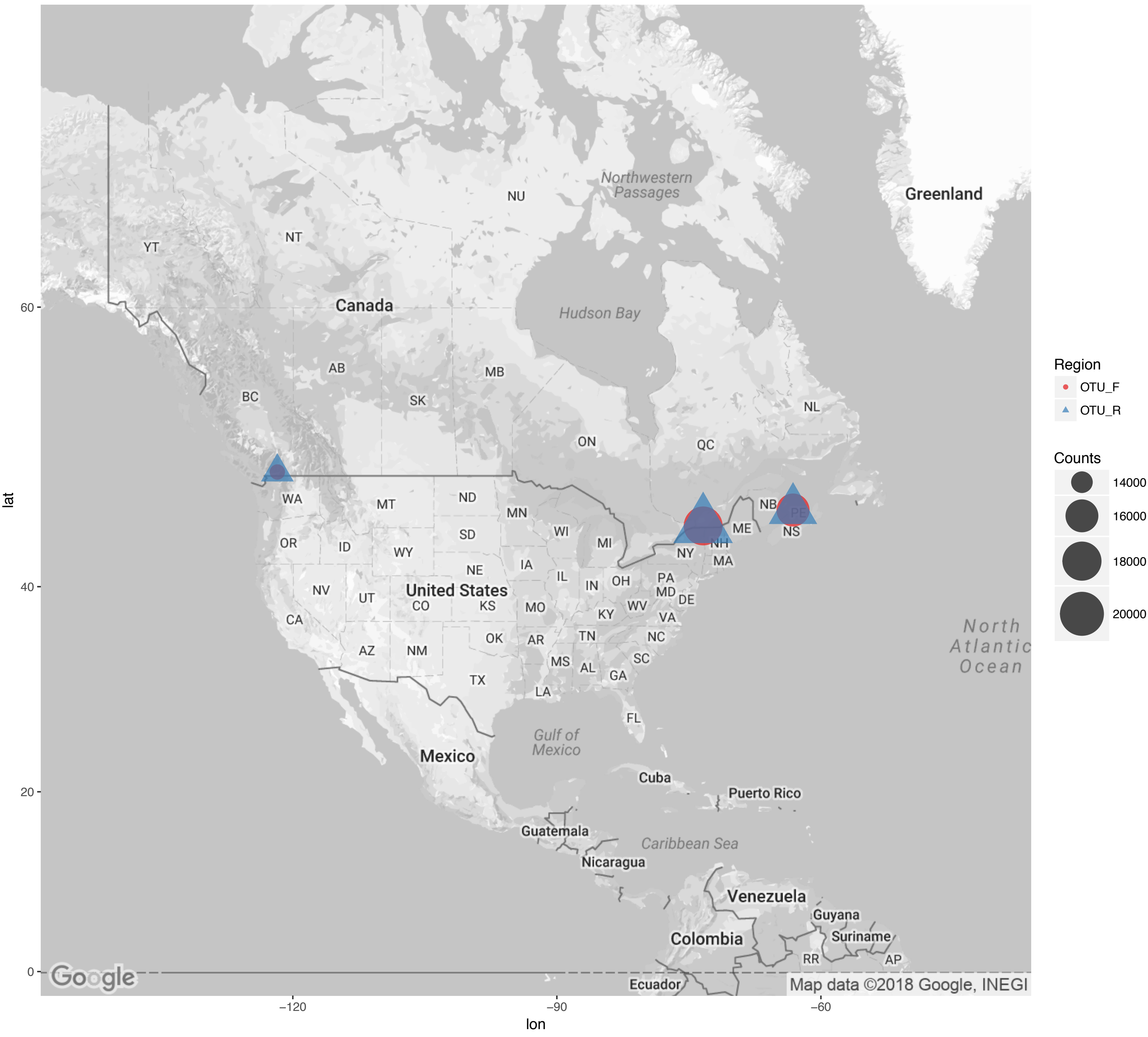

Fig. S1 Sampling locations and sequence counts for ITS1_OTU (red solid dots) and ITS2_OTU (blue solid triangle).

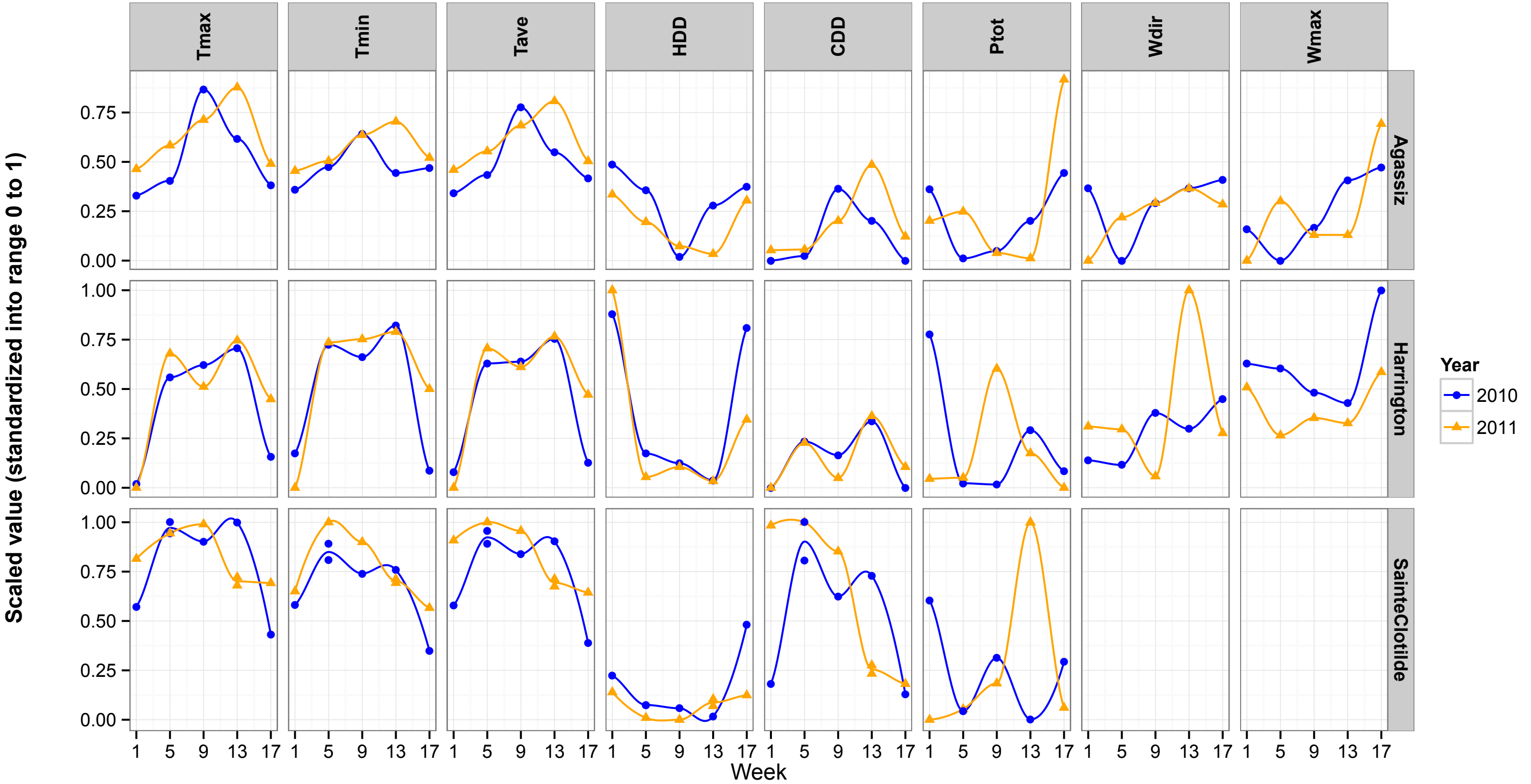

Fig. S2 Climatological data of sampling time points. The value of each variable was scaled to range 0 to 1. Tmax, maximum temperature; Tmin, minimum temperature; Tave, average temperature; CDD, Cooling Degree Days; HDD, Heating Degree Days; Ptot, total precipitation; Wdir, wind direction defined as the degree from north; Wmax, maximum wind speed. The wind data were missing for the sampling site at Saint−Clotilde (SC), Québec. The temperature-associated parameters assessed during the sampling weeks at each location were similar for 2010 and 2011, but the total precipitation and wind conditions were quite different. Ptot was higher at the beginning of the summer at all three locations, but during the summer, the east coast was wetter than the west coast. Wdir at Agassiz (west coast) was mainly from southwest to northeast (Wdir <= 90º from the north); while that of Harrington (east coast), was also mainly from southeast to northwest but occasionally from northwest to southeast (90 º <= Wdir <= 180 º from north). Wmax was much higher at Harrington than at Agassiz. Wind data were missing for the sampling site at Saint-Clotilde (east inland city); therefore, samples collected from this location were removed when wind data were included in a model.

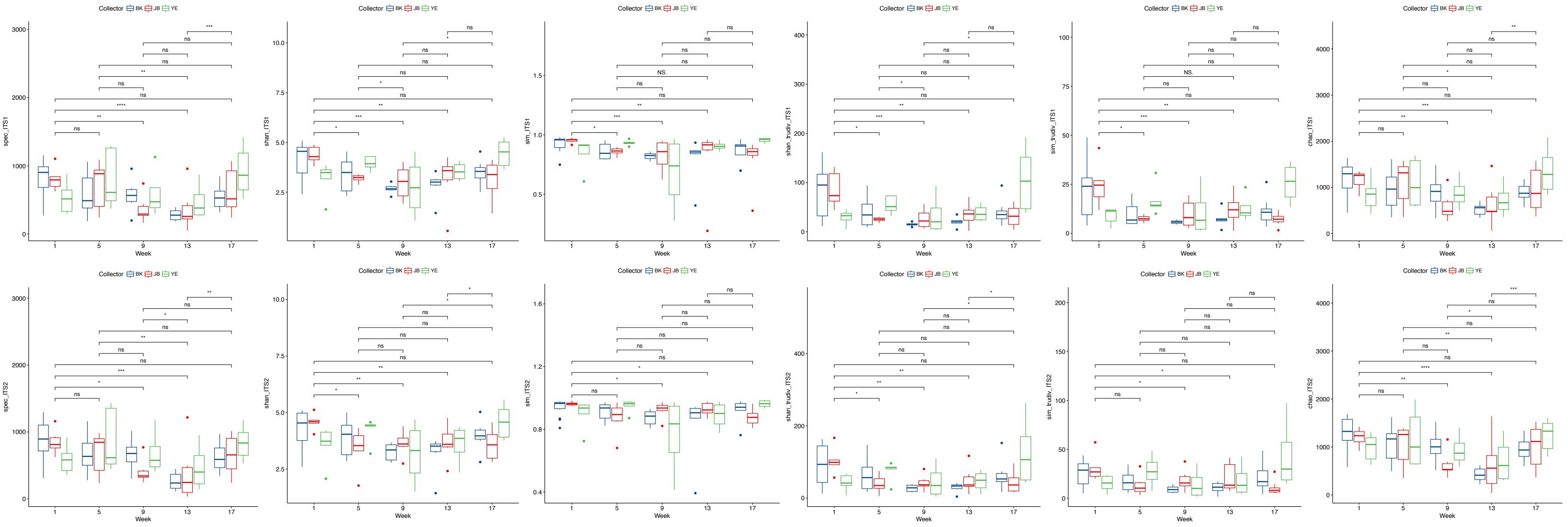

Fig. S3 The shift of diversity indices over the sampling weeks. Significant differences of the diversity indices between sampling weeks were annotated as: ***, p <= 0.001; **, p <= 0.01 and p > 0.001; *, p <= 0.05 and p > 0.01.

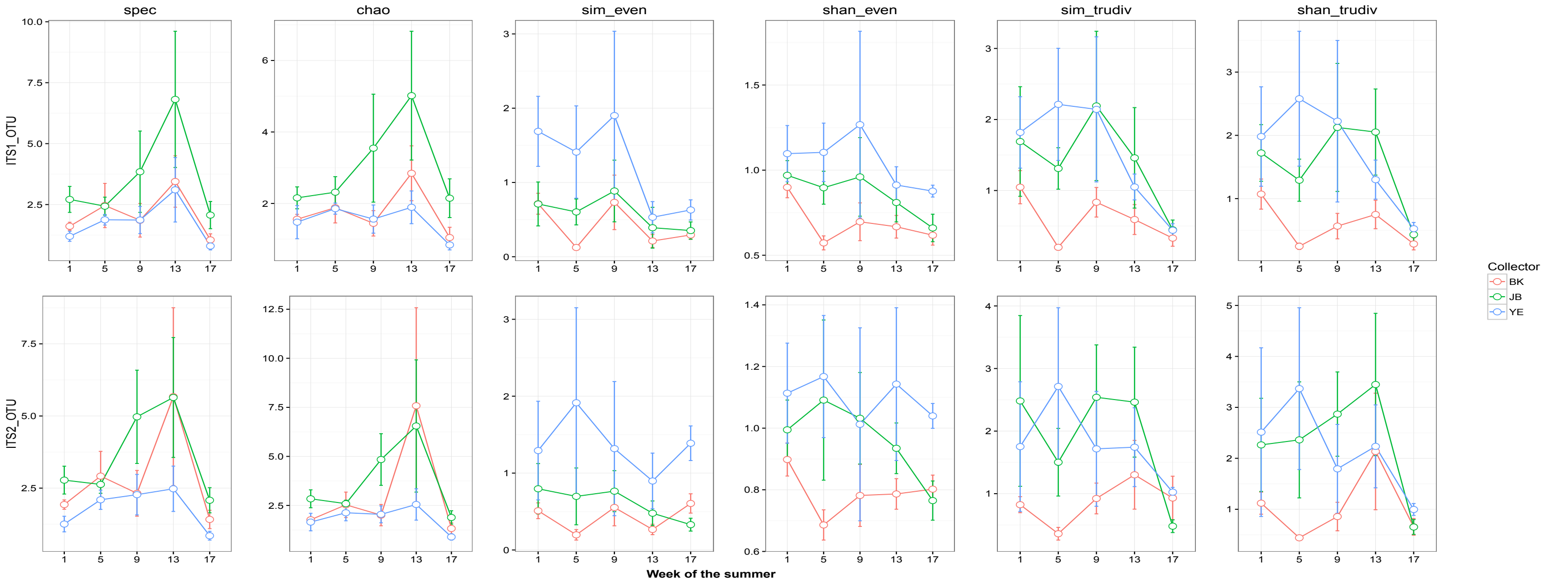

Fig. S4 The ratio of diversity indices of Ascomycota and Basidiomycota based on ITS1_OTU and ITS2_OTU. spec: specnumber (observed richness); chao: chao1 index (expected richness); sim_even: Gini-Simpson dominance evenness; shan_even: Shannon−Wiener evenness; sim_trudiv: Gini-Simpson true diversity; shan_trudiv: Shannon−Wiener true diversity.

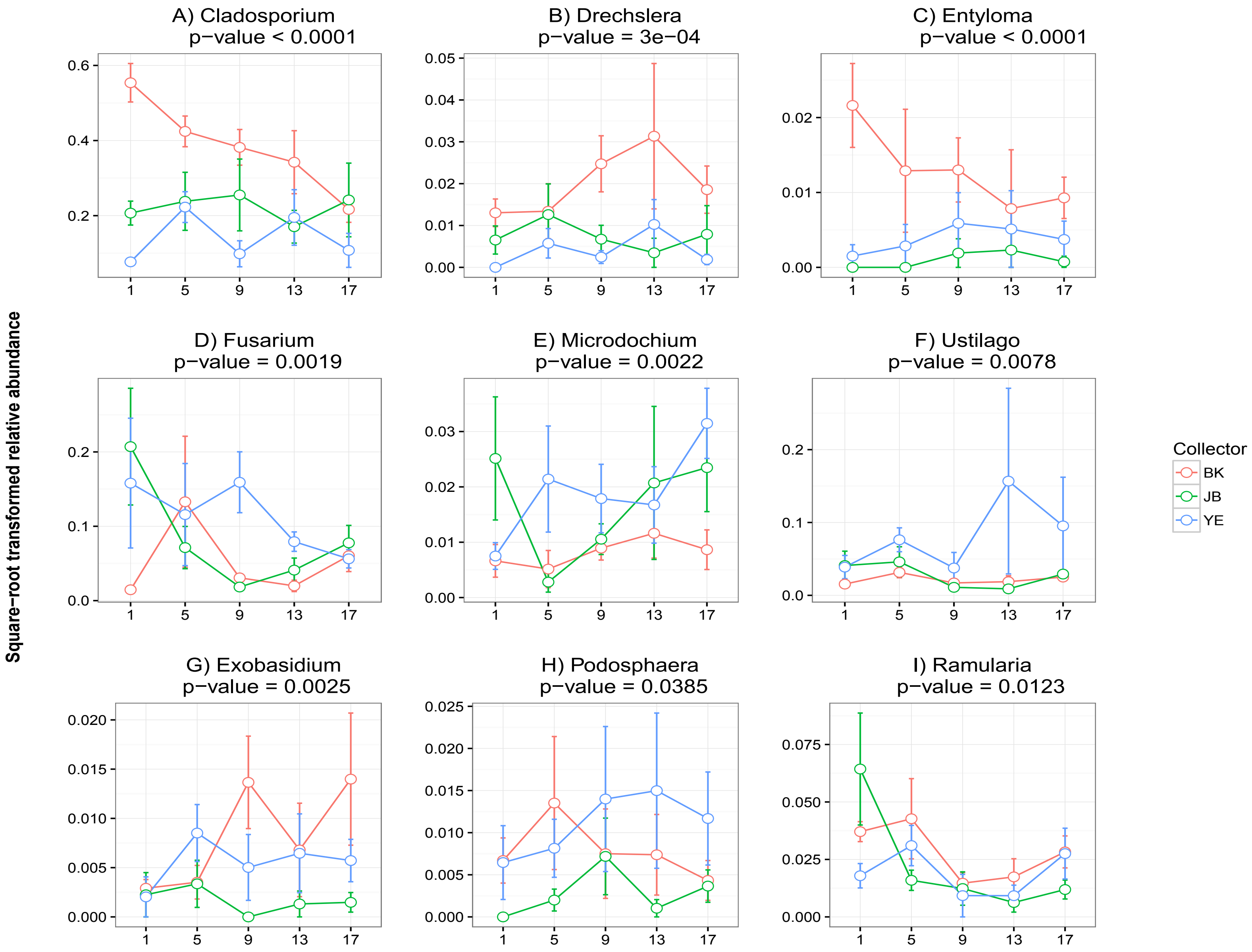

Fig. S5 The observed relative abundance of selected genera recovered by each spore sampler through the sampling season: a) *Cladosporium* spp., b) *Drechslera* spp., c) *Entyloma* spp., d) *Fusarium* spp., e) *Microdochium* spp., f) *Ustilago* spp., g) *Exobasidium* spp., h) *Podosphaera* spp., i) *Ramularia* spp.

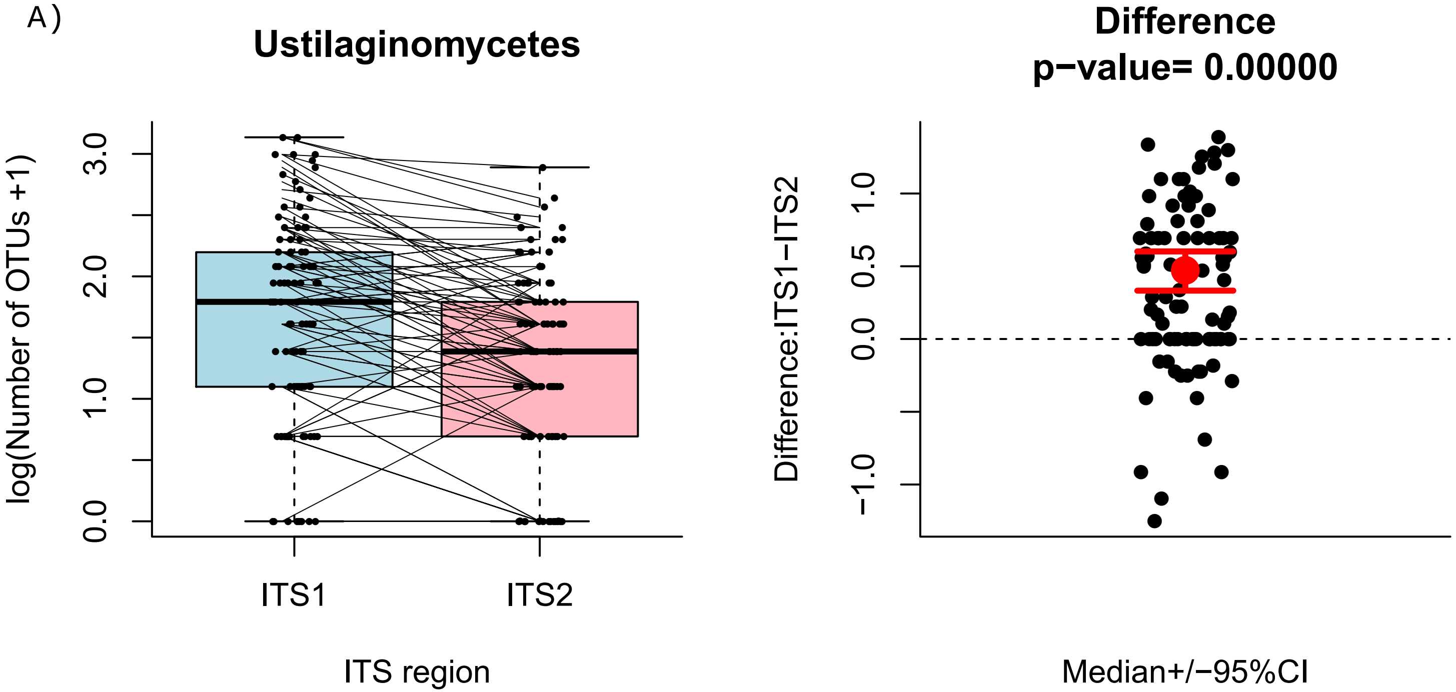

A)
Ustilaginomycetes
log(Number of OTUs +1)
0.0
1.0
2.0
3.0
ITS1
ITS2
ITS region
Difference
p−value= 0.00000
Difference:ITS1−ITS2
−1.0
0.0
0.5
1.0
Median+/−95%CI

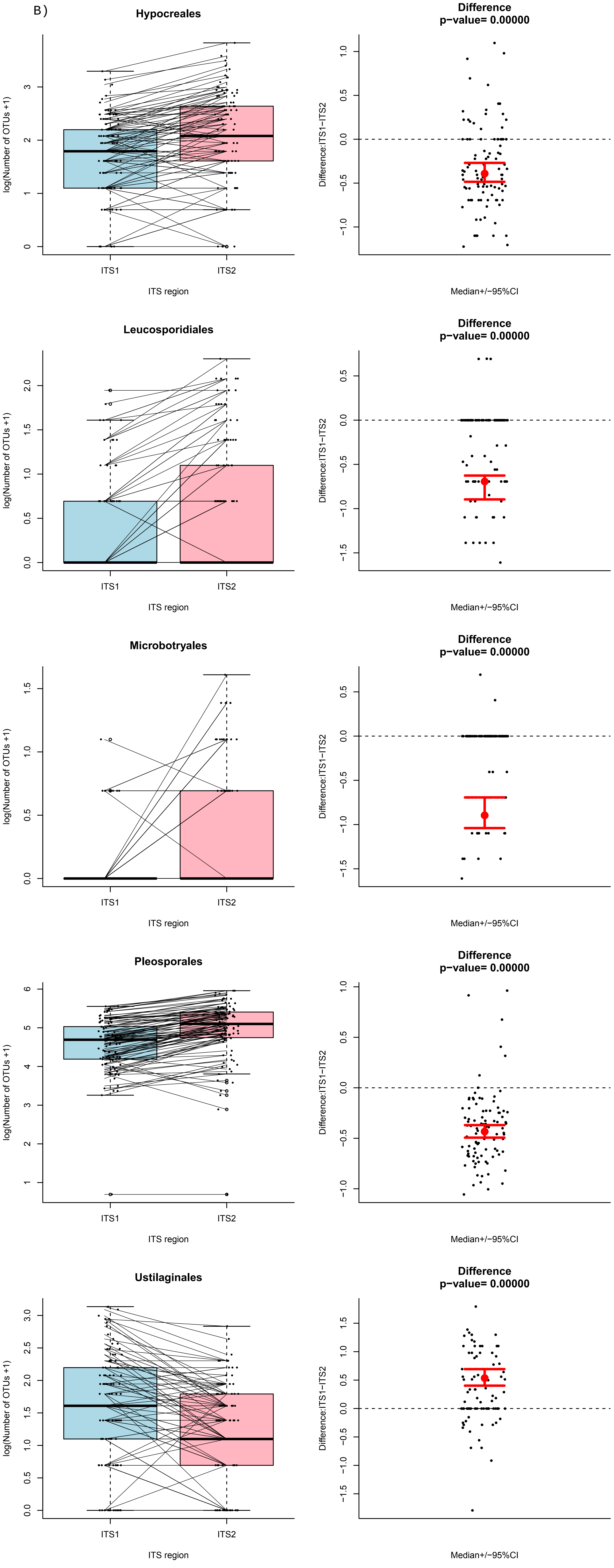
B)
Hypocreales
log(Number of OTUs +1)
ITS1
ITS2
ITS region
Difference
p−value= 0.00000
Difference:ITS1−ITS2
Median+/−95%CI
Leucosporidiales
log(Number of OTUs +1)
ITS1
ITS2
ITS region
Difference
p−value= 0.00000
Difference:ITS1−ITS2
Median+/−95%CI
Microbotryales
log(Number of OTUs +1)
ITS1
ITS2
ITS region
Difference
p−value= 0.00000
Difference:ITS1−ITS2
Median+/−95%CI
Pleosporales
log(Number of OTUs +1)
ITS1
ITS2
ITS region
Difference
p−value= 0.00000
Difference:ITS1−ITS2
Median+/−95%CI
Ustilaginales
log(Number of OTUs +1)
ITS1
ITS2
ITS region
Difference
p−value= 0.00000
Difference:ITS1−ITS2
Median+/−95%CI

c)

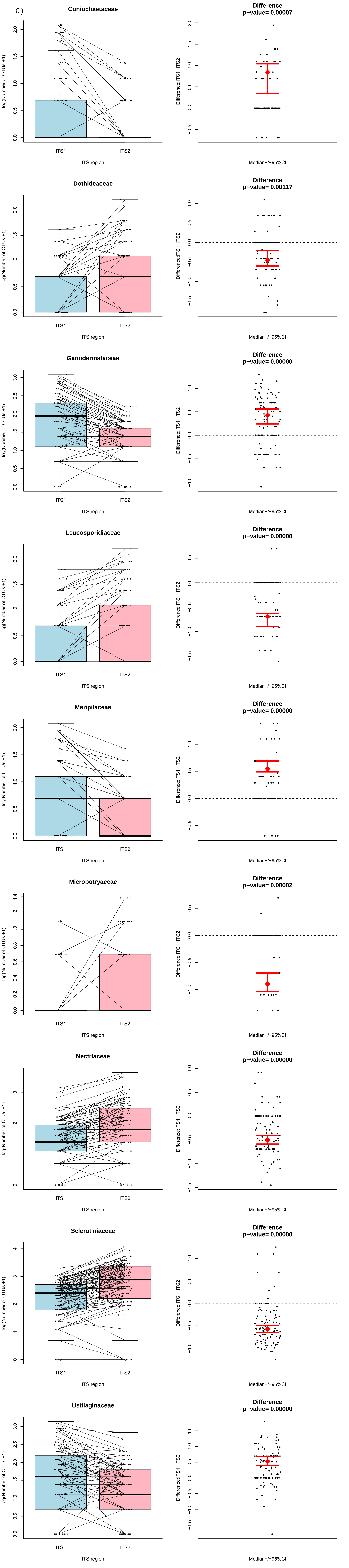
Coniochaetaceae
Difference
p−value= 0.00007
Dothideaceae
Difference
p−value= 0.00117
Ganodermataceae
Difference
p−value= 0.00000
Leucosporidiaceae
Difference
p−value= 0.00000
Meripilaceae
Difference
p−value= 0.00000
Microbotryaceae
Difference
p−value= 0.00002
Nectriaceae
Difference
p−value= 0.00000
Sclerotiniaceae
Difference
p−value= 0.00000
Ustilaginaceae
Difference
p−value= 0.00000
log(Number of OTUs +1)
ITS1
ITS2
ITS region
Difference:ITS1−ITS2
Median+/−95%CI

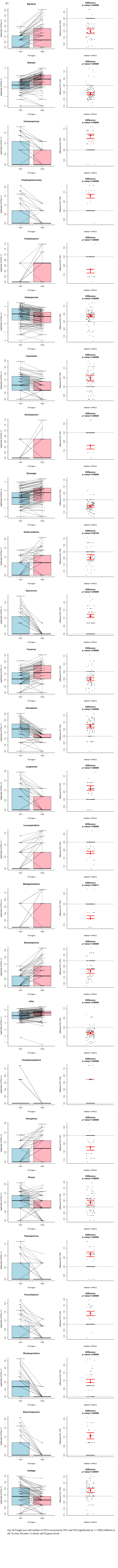

Fig. S6 Fungal taxa with number of OTUs recovered by ITS1 and ITS2 significantly (p <= 0.001) different at the A) class, B) order, C) family and D) genus levels.
